# True Preoperative Liquid Fasting in Romania—A Secondary Analysis of the Thirst Study

**DOI:** 10.3390/nu18111714

**Published:** 2026-05-27

**Authors:** Emanuel Moisa, Silvius Ioan Negoita, Anne Marie Camilleri Podesta, Daniela Ionescu, Dana Rodica Tomescu, Liliana Elena Mirea, Gabriela Droc, Bianca Liana Grigorescu, Cristina Petrisor, Alice Nicoleta Drăgoescu, Marius Bogdan Novac, Anca Irina Ristescu, Mihaela Blaj, Carmen Orban, Ovidiu Bedreag, Narcis Valentin Tănase, Madalina Dutu, Bogdan Ioan Vintila, Georgeana Tuculeanu, Federico Bilotta, Dan Corneci, on behalf of the Romanian Thirst Study Group

**Affiliations:** 1 Department of Anaesthesiology and Intensive Care, Carol Davila University of Medicine and Pharmacy, 050474 Bucharest, Romania; emanuel.moisa@umfcd.ro (E.M.); dana.tomescu@umfcd.ro (D.R.T.); liliana.mirea@umfcd.ro (L.E.M.); gabriela.droc@umfcd.ro (G.D.); carmen.orban@umfcd.ro (C.O.); tanasenv@yahoo.com (N.V.T.); madalina.dutu@umfcd.ro (M.D.); georgeana.tuculeanu@drd.umfcd.ro (G.T.); dan.corneci@umfcd.ro (D.C.); 2 Department of Anaesthesiology and Intensive Care, Elias Emergency University Hospital of Bucharest, 011461 Bucharest, Romania; 3 Department of Anaesthesia, Intensive Care and Pain Medicine, Mater Dei Hospital, MSD 2090 Msida, Malta; anne-marie.camilleri-podesta@gov.mt; 4 Department of Surgery, Faculty of Medicine and Surgery, University of Malta, MSD 2090 Msida, Malta; 5 Department of Surgery, Anesthesiology and Intensive Care, Iuliu Hatieganu University of Medicine and Pharmacy, 400347 Cluj-Napoca, Romania; daniela_ionescu@umfcluj.ro (D.I.); petrisor.cristina@umfcluj.ro (C.P.); 6 Prof. Dr. Octavian Fodor Institute of Gastroenterology and Hepatology, 400162 Cluj-Napoca, Romania; 7 Association for Research in Anesthesia and Intensive Care, 400162 Cluj-Napoca, Romania; 8 Department of Anaesthesiology and Intensive Care, Fundeni Clinical Institute, 022328 Bucharest, Romania; 9 Department of Anaesthesiology and Intensive Care, Clinical Emergency Hospital of Bucharest, 014461 Bucharest, Romania; 10 Department of Anaesthesiology and Intensive Care, George Emil Palade University of Medicine, Pharmacy, Science and Technology, Targu-Mures, 540142 Targu-Mures, Romania; bianca.grigorescu@umfst.ro; 11 Department of Anaesthesiology and Intensive Care, Emergency County Hospital of Cluj-Napoca, 400347 Cluj-Napoca, Romania; 12 Department of Anaesthesiology and Intensive Care, University of Medicine and Pharmacy of Craiova, 200349 Craiova, Romania; alice.dragoescu@umfcv.ro (A.N.D.); marius.novac@umfcv.ro (M.B.N.); 13 Department of Anaesthesiology and Intensive Care, Faculty of Medicine, Grigore T. Popa University of Medicine and Pharmacy, 700115 Iasi, Romania; anca.ristescu@umfiasi.ro (A.I.R.); mihaela.blaj@umfiasi.ro (M.B.); 14 Department of Anaesthesiology and Intensive Care, Regional Oncology Institute, 700483 Iasi, Romania; 15 Saint Spiridon Emergency County Hospital, 700361 Iasi, Romania; 16 Bucharest University Emergency Hospital, 050098 Bucharest, Romania; 17 Department of Anaesthesiology and Intensive Care, Faculty of Medicine, Victor Babes University of Medicine and Pharmacy, 300041 Timisoara, Romania; bedreag.ovidiu@umft.ro; 18 Department of Anaesthesiology and Intensive Care, Dr. Carol Davila Central Military Emergency University Hospital, 010825 Bucharest, Romania; 19 Faculty of Medicine, Lucian Blaga University of Sibiu, 550169 Sibiu, Romania; bogdan.vintila@ulbsibiu.ro; 20 Department of Anesthesiology, Intensive Care Medicine and Pain Medicine, Tor Vergata University of Rome, 00133 Rome, Italy; bilotta.fb@gmail.com

**Keywords:** preoperative fasting, prolonged fasting, clear fluids, elective surgery, liberal fasting, fasting practices, anesthesia, fluid fasting, perioperative medicine, guidelines

## Abstract

**Background**: The recently published Thirst study showed that prolonged preoperative fasting for liquids remains an unresolved issue across multiple European countries despite clear guideline recommendations. In-depth analyses of national practices could help the development of targeted interventions. The aim of our study was to provide a national overview of fluid fasting in Romania, highlighting institutional factors that could prolong two distinct fasting times: true fluid fasting time and fasting time until the last sips. **Methods**: This was a secondary analysis of the recently published Thirst study, a prospective, observational, multicenter study. Twenty-one Romanian centers recruited a total of 2185 adult patients undergoing elective procedures between 25 and 29 November 2024. The main outcomes were: the median value of self-reported fasting time to the last sips (SIPS) and true fluid fasting time for larger amounts (NOT SIPS) across centers and procedures; SIPS and NOT SIPS times across hospital level of care and fluid fasting protocols; and the independent predictive value of hospital level of care, fluid fasting protocols and workload for prolonged fasting. The secondary outcomes were the frequency of prolonged fasting (>4 h) and fasting for 2–4 h across centers, procedures, level of care, protocol and afternoon scheduling. The Kruskal–Wallis test with pairwise comparisons was used to compare differences in median times depending on the studied subgroup. **Results**: The median fluid fasting times for SIPS and true fluid fasting were 8 h [3:30–13:00] and 12 h [10:00–14:55], respectively, and varied significantly across centers, specialties, levels of care and protocol in place (*p* < 0.001). Prolonged SIPS and true fluid fasting times were observed in 67.3% and 95% of the patients, respectively. Both times varied significantly across the procedure types (*p* < 0.05), with ophthalmology having the shortest fasting times. Reduced fluid fasting times were independently associated with moderate-competence centers, lower emergency case load and guideline-based fasting protocols. **Conclusions**: This secondary analysis shows that non-adherence to fluid fasting guidelines is frequent in most Romanian regions and centers. Further research is needed to develop individualized strategies based on institutional and geographical policies as well as on physiologic effects of fluid fasting.

## 1. Introduction

It was not until the late 20th century that professional anesthesiology societies developed guidelines permitting the intake of clear liquids up to two hours before surgery, despite the *nil per os* after midnight practice [1]. Two decades later, many patients still fast for excessively long periods [2]. This is mainly attributable to organizational factors, including unpredictable operating room schedules, limited staff, unclear communication regarding fasting instructions, and the excessively restrictive interpretation of the concept of *nil per os* [2].

Prolonged fasting can lead to metabolic disturbances and can significantly increase patient discomfort and perioperative symptoms. Patients often experience thirst, hunger, anxiety, fatigue, nausea, vomiting, increased pain perception and emergence delirium [3]. Excessive fluid restriction can lead to serious systemic effects beyond discomfort, including cardiovascular, renal, and respiratory complications [1,4].

Quality improvement initiatives have been effective in bridging the gap between evidence-based fasting guidelines and real-world practices, concluding that prolonged preoperative liquid fasting is primarily an organizational and behavioral issue rather than a knowledge deficit [5]. Context-adapted interventions, such as staff education, standardized patient information, visual reminders, and individualized fasting cards, resulted in a sustained reduction in median liquid fasting time from approximately 12 h to nearly guideline-recommended levels of 2 h [2].

Despite the significant body of evidence and strong guideline recommendations supporting a 2 h fasting time for clear liquids, and even more liberal practices as per a recent consensus [6], the Thirst study showed that the implementation of this policy remains poor [7]. Notably, there was an important discrepancy between the written protocols at the institutional level and actual fluid fasting times. International data is essential, but an in-depth analysis at a national level, which could better characterize local variations in fluid fasting practices, could serve as a starting point for individualized and effective strategies.

We aimed to conduct a secondary analysis of the Thirst study, which is notable for being the first large-scale, multicenter Romanian study investigating fluid fasting practices across 21 national centers. In addition to the original Thirst study, our analysis is based on a clear distinction between true fluid fasting time and fasting time until the last “sips”, as this may constitute an important confounder, as well as a critical consideration when defining areas of concern and outcome measures within optimization strategies. Furthermore, we accounted for the fasting protocols in place, for the level of care provided by participating centers, considering the national grading scales, for emergency case volume, and for patient risk profile. Given that most effective local quality improvement initiatives have been based on large-scale prior audits [8,9], our findings may represent an initial step towards developing individualized strategies to enhance guideline adherence at a national and/or institutional level.

## 2. Materials and Methods

### 2.1. Study Design and Recruitment

This is a secondary analysis of the Thirst study [7]—a prospective, multicenter, observational study conducted in 46 centers from 12 European countries, endorsed by the European Society of Anaesthesiology and Intensive Care (ESAIC) and registered in ClinicalTrials.gov (NCT06527703). A national call was made in Romania to all heads of the anesthesiology and intensive care departments through the official email of the Romanian Society of Anaesthesiology and Intensive Care. Twenty-one public centers agreed to participate, and local coordinators were appointed.

### 2.2. Study Protocol and Procedures

The study documents were translated into Romanian by national coordinators and sent to local coordinators. Several sessions of online training were conducted, and all participating centers agreed on a working week (5 days, from Monday to Friday) in which recruitment took place (25–29 November 2024), as per the study protocol. For the original study, all centers obtained approval from their Local Ethics Committee. For this secondary analysis, a waiver was granted by the Local Ethics Committee from the Elias Emergency University Hospital of Bucharest, Romania (Reference number: 25112025-1, Date: 25 November 2025), as no additional data about patients were collected, and patients’ data for the original Thirst study were collected anonymously. Each center reported additional data related to their corresponding center (such as total number of anesthesiologists, theatres, procedures, etc.). All research was conducted in accordance with the ethical guidelines of the World Medical Association Declaration of Helsinki.

### 2.3. Study Population and Data Collection

No deviation from the original study protocol was made, which is available in the Supplementary Materials of the Thirst Study [7]. Briefly, adult patients requiring general or regional anesthesia or sedation for elective procedures were recruited in this study. Informed consent was obtained in the operating room or holding bay, without previous notice. After consent was obtained, patients were given a unique code and completed a self-reported questionnaire in which they completed two fluid fasting times. The definitions of these fluid fasting times were explained to all participants.

### 2.4. Outcomes and Definitions

#### 2.4.1. Primary Outcome

A. SIPS and true fluid fasting time (NOT SIPS) times across centers and procedures.
B. SIPS and true fluid fasting time across hospital level of care and fluid fasting protocols.
C. Independent predictive value for prolonged fasting of hospital level of care, fluid fasting protocols and workload.

Definitions:

A. The SIPS time was defined as the interval between the procedure and the last ingestion of a very small volume of clear liquid. In the Romanian context, this most commonly reflected fluid intake solely to facilitate the administration of morning medications and did not represent intentional preoperative hydration, except for some centers reporting local protocols in which sips of clear liquids are allowed or encouraged preoperatively [7] (Supplementary File S1).
B. True fluid fasting time (NOT SIPS) was defined as the interval between the procedure and the last ingestion of clear liquids, excluding negligible volumes taken as sips.
C. Hospital category was defined according to the national classification of hospitals as per the Ministry of Health from Romania [10]: (1) Category Ia hospitals—maximum level hospitals (regional referral hospitals with the highest level of specialization, staffing, infrastructure, and complexity of care; usually institutes, including transplantation centers); (2) Category Ib—very high level of competence (regional or county referral hospitals with a high level of specialization, staffing, infrastructure, and complexity of care; does not include institutes or transplantation centers); (3) Category II—high competence (County/regional hospitals managing complex cases with broad specialty coverage); (4) Category III—moderate competence (general hospitals managing conditions of intermediate complexity).
D. Workload was defined based on three variables: total number of anesthesiologists per center, total number of theaters (operating rooms and theaters for procedures outside the operating rooms) and total number of procedures/year. For this study, all centers reported their data for the year 2024, given that the original study was conducted in November 2024. Three ratios were derived as follows: Anesthesiologist-to-Theater ratio (Total number of Anesthesiologists/Total number of theaters), Procedures-to-Theater ratio (Total number of procedures per year/Total number of theaters) and Procedures-to-Anesthesiologist ratio (Total number of procedures per year/Total number of Anesthesiologists). As in Romania, anesthesiologists also rotate in the intensive care unit; the total number of anesthesiologists represented the effective number of anesthesiologists distributed to theaters. Moreover, the percentage of emergency procedures was reported from every center.

#### 2.4.2. Secondary Outcomes

A. Frequency of preoperative SIPS and NOT SIPS times of <2 and 2–4 h.
B. Prolonged fasting rates across centers, procedures, hospital category, protocol in place and afternoon scheduling. This was defined as the percentage of patients who fasted (NOT SIPS time) for >4 h before the procedure, based on previous reports [8,11,12].

### 2.5. Statistical Analysis

Statistical analysis was conducted using IBM Statistical Package for Social Sciences (SPSS) for Windows, version 30.0 (IBM Corp., Armonk, NY, USA). Data were tested for distribution normality using the Kolmogorov–Smirnov test with Lilliefors correction and visual representation. Outlier analysis was performed, and each case was studied for possible error in data entry. No outliers were removed, as similar outliers were reported in different centers in this study, and this was considered significant for the present analysis. All continuous data followed a nonparametric distribution and were reported as medians and interquartile range [25–75%]. Comparisons between two independent groups were performed using the Mann–Whitney U test. For three or more independent groups, the Kruskal–Wallis H test with Bonferroni correction was used for multiple comparisons. Given the high number of groups studied, heat maps were generated to highlight the significant average rank differences across the groups. Categorical data were expressed as absolute (number) and relative (percentage) frequencies, and Chi-square or Fisher exact tests were used accordingly. Mixed linear models with fixed and random effects were performed to test the effect size of hospital level of care, workload, fluid fasting protocols, gender and afternoon scheduling on fluid fasting times (dependent), considering center as a random effect. The full models were reported, and the estimates of effect size were plotted as forest plots. Scatter plots with fit lines were used for visual exploration of linear associations. Binary logistic multivariable regressions were conducted to test the independent predictive value for prolonged fasting of all possible confounders. An alpha level < 0.05 was considered significant, and all tests were two-tailed.

This manuscript was prepared in accordance with the EQUATOR (Enhancing the QUAlity and Transparency Of health Research) guidelines using the STROBE (Strengthening the Reporting of Observational Studies in Epidemiology) checklist (Supplementary File S2). Table editing in Supplementary File S3 was done with the help of artificial intelligence tools (ChatGPT, version 5, OpenAI, 25 January 2026) based on SPSS-generated output tables. There was no intervention on the statistical findings in the process.

## 3. Results

A total of 2185 patients from 21 public (18 academic and three non-academic) Romanian centers were included. Procedure distribution was uneven and comprised mainly general surgery (624/2185), endoscopy (427/2185), orthopedic surgery (250/2185) and obstetrics–gynecology (225/2185), while non-operating room anesthesia (NORA, 43/2185) and cardiothoracic (34/2185) procedures had the lowest representation. One hundred and thirty-nine patients requiring regional or general anesthesia for ophthalmological procedures were recruited from a single center. The complete distribution of patients per center and specialty is reported in Supplementary Material File S3.

### 3.1. SIPS and True Fluid Fasting (NOT SIPS) Times Across Centers

#### 3.1.1. SIPS Times Across Centers

**Figure 1:**
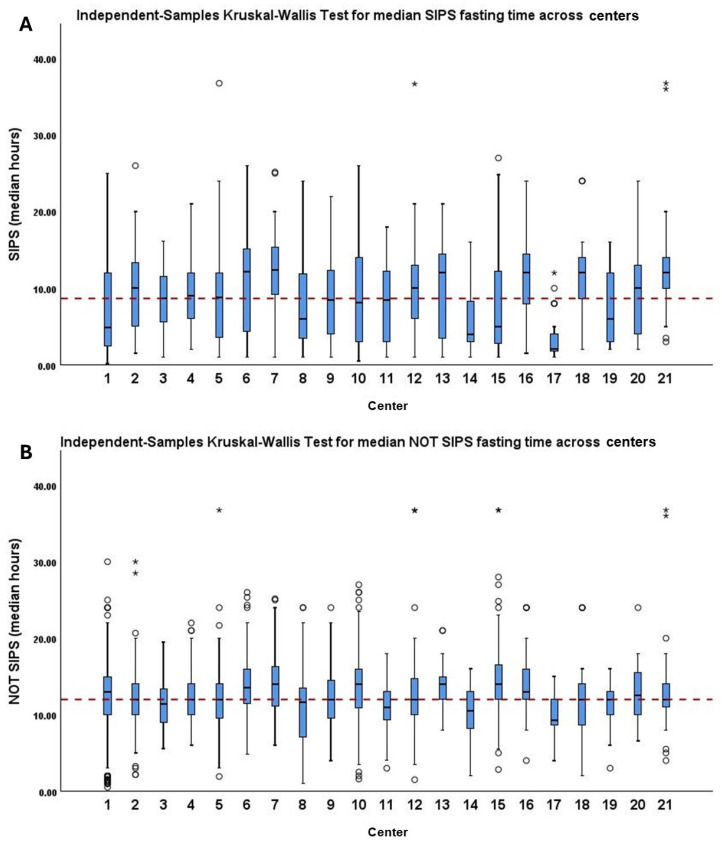
SIPS (**A**) and true fluid (NOT SIPS) (**B**) median fasting times across centers. Boxplots represent median fasting times with interquartile range. The red dashed line represents the median of the whole sample. Centers are presented in anonymized form. The figure illustrates the variability in fasting practices across participating centers. Both times varied significantly across centers. Pairwise comparisons with Bonferroni correction are reported in Supplementary File S3. Circles and asterixis represent subjects outside the interquartile range.

The median SIPS time in the whole cohort was 8 h [3:30–13:00], with a minimum of 10 min and a maximum of 36 h and 45 min, respectively. The median SIPS time varied significantly across centers (*p* < 0.001), with one center reporting the lowest median time of 2 h and 5 min [1:44–4:00] and six centers reporting median SIPS times between 12 h and 12 h and 20 min [3:30–15:23] (Figure 1A). Centers 1, 5, 8, 9, 11, 14, 15, 17 and 19 had significantly lower median times than other centers.

#### 3.1.2. True Fluid Fasting (NOT SIPS) Times Across Centers

The median NOT SIPS time was 12 h [10:00–14:55], with a minimum of 30 min and a maximum of 36 h and 45 min, respectively. NOT SIPS time varied significantly across centers (*p* < 0.001), with one center reporting the lowest median time of 9 h and 15 min [8:40–12:00] and four centers reporting the longest median times of 14 h [10:49–16:34] (Figure 1B). A detailed analysis of all median SIPS and NOT SIPS times across centers is reported in Supplementary Material File S3.

#### 3.1.3. SIPS and True Fluid Fasting (NOT SIPS) Time Across Academic Versus Non-Academic Centers

Only 145 patients were recruited from three non-academic centers, with the remaining 2040 patients from 18 academic centers. SIPS and true fluid fasting times were significantly shorter in non-academic centers: 6 [2:30 to 12:15] vs. 8 h [3:30 to 13:00] for SIPS time (*p* = 0.021) and 12 [9:52 to 14:00] versus 12 h and 20 min [10:00 to 13:00] for true fluid fasting (*p* = 0.037), respectively.

### 3.2. SIPS and True Fluid Fasting (NOT SIPS) Times Across Specialties

#### 3.2.1. SIPS Across Specialties

**Figure 2:**
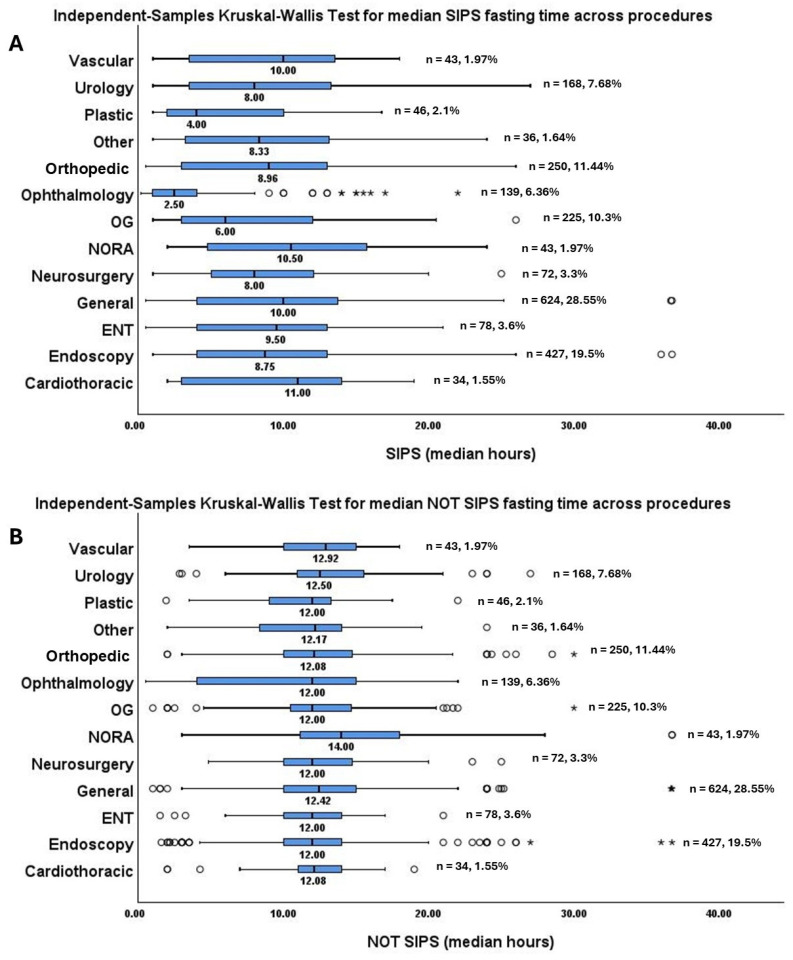
SIPS (**A**) and true fluid (NOT SIPS) (**B**) median fasting times across specialties. Boxplots represent median fasting times with interquartile range. Both times varied significantly across specialties. Pairwise comparisons with Bonferroni correction are reported in Supplementary File S3. Circles and asterixis represent subjects outside the interquartile range.

Significant differences were observed across specialties for SIPS time (*p* < 0.001), with ophthalmology having the lowest median time of 2 h and 30 min [1:00–4:00], while NORA and cardiothoracic surgery had the longest median times of 10 h and 30 min [4:30–16:15] and 11 h [3:00–14:00], respectively (Figure 2A). Ophthalmology had a significantly lower median time than all other specialties (*p* < 0.001), except plastic surgery (*p* > 0.05). Moreover, significantly shorter times were observed for plastic surgery compared with endoscopy, general surgery and NORA (*p* < 0.05) and between obstetrics–gynecology (OG) and general surgery (*p* = 0.008). All other pairwise comparisons were not statistically significant (*p* > 0.05).

#### 3.2.2. True Fluid Fasting (NOT SIPS) Times Across Specialties

Significant differences were observed across specialties for NOT SIPS time (*p* < 0.001), although all median times fell within a narrow range—between 12 and 13 h, except for NORA (median time 14 h [11:00–18:00] (Figure 2B). The only significant differences were observed between ophthalmology and general surgery, and urology and NORA (*p* < 0.05).

#### 3.2.3. SIPS and True Fluid Fasting (NOT SIPS) Times Across Specialties Between Centers

The SIPS time across centers was significantly different among the following specialties: endoscopy, general surgery, neurosurgery, NORA, OG, orthopedic, other procedures, and urology.

The NOT SIPS time across centers was significantly different among the following specialties: endoscopy, ear–nose–throat (ENT), general surgery, NORA, OG, orthopedic and urology. All median SIPS and NOT SIPS times, as well as pairwise comparisons, are available in Supplementary File S3.

### 3.3. Hospital Level of Care (Category), Workload, Fasting Protocols, Afternoon Schedule and Fasting Times

#### 3.3.1. Hospital Category, Workload and Fasting Protocols

Hospital category was defined in the Section 2. Patients had an uneven distribution, with category Ib being the most represented (1096/2185, 50.16%), followed by category II, Ia and III hospitals, which included 499/2185 (22.84%), 367/2185 (16.8%) and 223/2185 (10.2%) patients, respectively (Table 1). Median number of anesthesiologists, theaters (operating rooms and non-operating room anesthesia theaters) and procedures/year differed significantly depending on hospital category, with category Ib hospitals having the highest median number of anesthesiologists, theaters and procedures (*p* < 0.001) (Table 1). Three indices of workload were calculated based on the number of anesthesiologists, theaters and procedures. Anesthesiologists-to-theater ratio was significantly different (*p* < 0.001), with category II hospitals having the lowest median ratio of 0.63 [0.63 to 1], followed by category Ib, III and Ia with median ratios of 0.65 [0.41 to 0.85], 0.7 [0.5 to 0.7] and 0.75 [0.61 to 0.75], respectively. For procedures-to-theater and procedures-to-anesthesiologists ratio, category Ib hospitals had the highest median values of 508.02 [508.02 to 610.05] and 986.84 [625 to 1225.24], respectively. Category III hospitals had the lowest median ratio values of 250 [150 to 335.8] and 300 [300 to 479.7], respectively. Lastly, category Ia hospitals had the lowest median rate of emergency procedures/year (13.35% [0.45% to 35%]), while in category III hospitals, we reported the highest median rate (33.4% [22.2% to 33.4%]), *p* < 0.001 (Table 1).

In terms of fluid fasting protocols, a guidelines-based approach was mainly used in category Ib and II hospitals, while category Ia hospitals allowed fluids until morning regardless of procedure time more frequently. Lastly, an NPO after midnight protocol was mainly followed in category III hospitals (*p* < 0.001). All data presented in this section are summarized in Table 1 and in Supplementary Material File S1.

#### 3.3.2. SIPS and True Fluid Fasting (NOT SIPS) Times Across Hospital Categories, Fasting Protocols and Afternoon Schedule

Both SIPS and NOT SIPS median times differed significantly (*p* < 0.001) across hospital categories, with category III hospitals having the shortest times (SIPS median 5 h [3:00 to 12:00] and NOT SIPS median 11 h [9:00 to 13:00]), while category II had the highest fluid fasting times (SIPS median 10:20 h [4:00 to 14:00], NOT SIPS median 13:30 h [11:00 to 15:30].

The SIPS median time was significantly shorter in patients from centers following a guidelines-based protocol compared to an NPO after midnight approach (8 [3:00 to 13:00] versus 9 h [4:00 to 12:30], *p* = 0.049), but it was longer in patients from centers that have an afternoon schedule (8 [3:15 to 10:00] versus 10 h [4:00 to 13:00], *p* = 0.025). Median NOT SIPS time was significantly different (*p* < 0.001) across all three protocols, as follows: 12 h [10:00 to 14:00] (NPO after midnight), 12:30 [10:00 to 14:00] (fluids allowed until morning) and 12:20 [10:00 to 14:40] (guidelines-based). No differences in NOT SIPS times were observed for centers having an afternoon schedule (*p* = 0.805).

### 3.4. Linear Mixed Models with Fixed and Random Effects

Three linear mixed-effects models were developed with fluid fasting time as the dependent variable, center as a random effect, and the workload indices (one in each model because of high multicollinearity): anesthesiologist-to-theatre ratio, procedures-to-theatre ratio and procedures-to-anesthesiologist ratio, together with hospital category, protocol in place, afternoon schedule and gender as fixed effects. All models are reported in Supplementary Material File S4.

Although workload indicators were associated with fasting time in some centers, the direction and magnitude of these associations were inconsistent across institutions (Figures S4.1–S4.3). For example, centers with relatively unfavorable workload ratios occasionally demonstrated shorter fasting times, whereas some centers with more favorable workload ratios exhibited prolonged fluid fasting.

Scatter plots were used for visual explorations. For all three workload indices, a non-linear distribution was observed. Fit lines were applied to test the possible non-linear interaction. For all three indices, a weak cubic relation was observed, suggesting that workload indices alone are poorly associated with prolonged fluid fasting (Supplementary Material File S4, Figures S4.1–S4.3).

### 3.5. Frequency of SIPS and NOT SIPS Times of <2 and 2–4 h

Overall, 7.5% (164/2185) and 0.8% (18/2185) of patients had SIPS and NOT SIPS times of <2 h, respectively. Notably, 11/18 patients who fasted (NOT SIPS) for <2 h underwent an ophthalmology procedure. Only the SIPS time of <2 h varied significantly across centers and regions (*p* < 0.05).

SIPS and NOT SIPS times of 2–4 h were observed in 25.2% (550/2185) and 4.2% (91/2185) of patients, respectively. Both times varied significantly across centers, hospital categories and fluid fasting protocol (*p* < 0.05). No difference was observed for centers that have an afternoon schedule (*p* > 0.05). A complete description of these findings is available in Supplementary File S5.

### 3.6. Prolonged Fasting

The overall prolonged fasting rate was 67.3% (1471/2185) for SIPS and 95% (2076/2185). Prolonged fasting differed across procedure type, hospital categories, fluid fasting protocols and centers with an afternoon schedule. The full analysis is reported in Supplementary File S6.

### 3.7. Binary Logistic Regression to Test the Independent Association with Prolonged Fluid Fasting

Two binary logistic multivariable regression models were constructed to test the independent association with prolonged fasting of hospital category, fluid fasting protocol, percentage of emergency procedures, afternoon schedule and gender. The dependent variable was the frequency of NOT SIPS or SIPS times > 4 h. Both models are reported in Table 2 and Table 3.

For true fluid fasting (NOT SIPS), category II hospitals and the percentage of emergency surgeries were significantly associated with prolonged fasting, while a guidelines-based protocol was associated with a lower frequency of prolonged fasting (Table 2).

For SIPS time, the same association was observed with a higher frequency of emergency surgery, while category III hospitals and a guidelines-based or fluids until morning approach were associated with a decreased frequency of patients having SIPS times > 4 h (Table 3).

## 4. Discussion

This study is a secondary analysis of the recently published Thirst study [7]. It provides a national snapshot of preoperative fluid fasting practices across 21 centers in Romania. The median true fluid fasting time (NOT SIPS) was 12 h—matching the Thirst cohort, while the median time to the last sips (SIPS) was 8 h. Both times showed inter-center and procedure-related variability but overall followed the same patterns, that is, excessive fluid fasting. The reported national prolonged fasting rate (>4 h) was 95%, while a 2 to 4 h fasting time was found in only 4.2% of patients. Patients from centers classified as III in terms of level of care, centers where fewer emergency procedures were performed and centers with protocols aligned to current guidelines had lower rates of prolonged fasting. Our findings underscore a persistent gap between guideline recommendations and real-world practices, stressing that barriers to adherence—likely related to emergency workload and educational background—remain unresolved in Romania, as well as across many European countries [7].

This is the first multicentric study of national fluid fasting practices on a sizeable cohort, with considerable center- and procedure-related variability, which is mainly representative of academic institutions providing high levels of care. Due to the unbalanced contribution of different countries to the Thirst cohort, with Romania providing data from 21/46 centers and 2185/5100 patients, this study adds important context to the original Thirst findings [7]. Therefore, this secondary analysis extends the original Thirst Study by introducing a more nuanced assessment of fluid fasting behavior, distinguishing between true fasting and intake of minimal volumes (“sips”). In addition, the national-level assessment enabled consideration of center-related characteristics, including workload, level of care and local protocols, thereby supporting hypothesis generation. Hence, focusing on a single national cohort favors a more detailed evaluation of system-level practices, variability, and implementation gaps that may be obscured in multinational analyses, thus providing actionable data to inform national quality improvement initiatives to reduce excessive preoperative fasting.

In terms of specialty, significantly longer fasting times were reported before NORA procedures, whereas ophthalmology consistently showed significantly shorter median fasting times than other specialties. Numerous centers reported same-day preanesthetic evaluation with NORA procedures, which could partially explain these results. However, a recent meta-analysis highlighted the increased patient satisfaction and safety of a non-fasting policy prior to cardiac catheterization [13]. Ophthalmology median SIPS time was significantly shorter; this subgroup warrants a separate consideration, since all 139 ophthalmology cases included in this analysis (as well as in the original Thirst cohort) originated from a single center, making these observations susceptible to local protocols rather than to a generalized practice. Evidence focusing on this population is limited. A study on 250 patients, mostly undergoing cataract surgery, reported a long mean fluid fasting time of 11.54 h [14], while a randomized controlled trial found that a preoperative non-fasting strategy is associated with less anxiety and surgical pain [15]. Therefore, for now, our results should be regarded cautiously pending validation in larger, multi-center designs.

One of the main strengths of the Thirst protocol, and consequently of this analysis, is the clear delineation between the true fluid fasting time (excluding the intake of a few sips of clear liquids) and fasting time to the last sips. The observed true fluid fasting time of 12 h lies close to the upper limit of the 9 to 13 h range reported in the available literature [14,16,17,18,19,20,21,22]. However, time to the last sips of clear fluids was rarely considered among the studied outcomes. Stressing the importance of this aspect, one iterative quality improvement study noted during their Plan-Do-Study-Act (PDSA) cycles discrepancies between true fluid fasting time and fasting time to the last sips [2], which aided further interventions. Of note, the most effective initiatives for reducing excessive fluid fasting are based on this strategy—PDSA cycles [2,5,23,24]. It seems that combining a more liberal fasting policy with iterative audits, education and reinforcement could lead to significant improvements in time [5].

Considering that a significant proportion of centers reported a *nil per os* after midnight protocol (most for both solids and fluids, but a few allow fluids until morning) for procedures scheduled before noon (accounting for >75% of cases in most centers), the discrepancy likely reflects the small volumes administered with morning medication rather than substantial preoperative hydration. Although we acknowledge the limitations inherent to an observational study design and do not infer causal relationships, a distinction between these two intervals seems clinically plausible since their implications may differ: prolonged true fasting time could contribute to preoperative hypovolemia and intraoperative hemodynamic disturbances, while prolonged SIPS time could mostly reflect comfort-related outcomes—thirst intensity and postoperative nausea and vomiting. Therefore, each fasting time probably warrants further research and different targeted interventions.

Several studies have investigated liberal protocols for preoperative fluid fasting. Allowing sips of water up until being called for surgery was found to be safe and effective in reducing fasting time as well as anxiety, thirst, nausea and vomiting [5,25]. In this national cohort, only 0.8% of patients fasted (NOT SIPS) for less than 2 h. Specific populations, such as pregnant patients and orthogeriatric patients, who are particularly susceptible to perioperative dehydration and neurocognitive deterioration, could benefit from this approach without adverse events nor a critical amount of residual gastric contents [25,26,27]. Offering a popsicle preoperatively has recently been validated as a strategy to decrease fluid fasting, as well as thirst, hunger and anxiety [28]. These findings support the implementation of standardized fasting protocols that allow clear fluids until call to theater (including popsicles, lemonade [27], etc.), supported by preoperative checklists, consistent patient instructions, and staff training to improve adherence and reduce unnecessary fasting.

The low frequency of patients fasting for 2 to 4 h (4.2%) and the very high prolonged fasting rate (95%) reported in our study are in line with results of similar studies [19,20,21]. Poor adherence to fasting guidelines is widespread, and the causes are multifactorial and related to patients, staff and institutional workflow. Some authors noted patients’ adherence to directives founded on personal research, as well as their reluctance to informal written instructions, pending verbal recommendations from their attending physician/nurse [5]. Poor compliance of health personnel (both surgical and anesthesia practitioners) with enforcing guideline-based recommendations remains a major barrier [29,30]. Interference of fasting times with surgical scheduling has been cited as one of the most important reasons for keeping patients fasted longer [5]. Educating staff on the adverse effects of prolonged fasting and improved patient–staff communication tends to decrease excessive fasting times [2,31].

In an effort to determine causal factors, our analysis included several center-level characteristics, such as level of care (according to the national classification), workload indices, emergency case load and the type of institutional fasting protocols in place. We observed that patients treated in moderate-competence institutions, in centers with a lower proportion of emergency cases, and in those with guideline-based fasting protocols in place were less likely to experience prolonged fasting, even when adherence to these protocols was inconsistent. Other authors also noted similar aspects, i.e., increasing fasting times with high volume of emergency procedures leading to rescheduling [2], superior adherence to guidelines where patient education is prioritized [32], a scenario compatible with centers with lower workload, where patient–staff interaction may be more consistent. Regarding workload indices, the linear mixed-effects models showed inconsistent and possible poor non-linear associations with fluid fasting times. These findings suggest that fasting duration may not be adequately explained by structural workload measures alone and that other factors may be involved: institutional culture, protocol adherence, educational interventions, scheduling efficiency, and communication practices.

This secondary analysis is subject to the same limitations as the original Thirst cohort. Notably, a few other aspects are worth mentioning. We acknowledge that our outcomes are based on patient self-reported fasting intervals, which are subject to recall bias, misinterpretation, and inaccurate estimation, particularly in older or anxious patients. The lack of objective verification (e.g., clinical records or timestamps) further limits data validity. Moreover, the absence of a standardized definition of “sips” and of a specified volume threshold may have introduced inter-center variability and misclassification, while age-related recall differences and potential underreporting may have further impacted accuracy. The predominance of patients from academic centers may introduce sampling bias and limit the generalizability of the findings to non-academic centers. Another limitation that biases the clinical relevance of the results on SIPS times is related to the type of protocols in place. A significant proportion of Romanian centers followed a *nil per os* after midnight approach [7], allowing sips of clear fluids with morning medication and not as a strategy meant to prevent or alleviate dehydration or other subjective symptoms. Thus, as we did not collect data on outcomes, the discussion of the effects of SIPS versus NOT SIPS (true fluid fasting) times on subjective versus physiological outcomes remains to be assessed in future studies. Furthermore, a stratification of patients based on age, American Society of Anesthesiologists (ASA) risk score and comorbidities (particularly cognitive and neurological impairment), which is currently lacking, could inform future targeted interventions and increase the validity of the linear mixed models. Also, more details on procedural complexity (e.g., minor versus major surgeries, anesthetic technique), the exact time of surgery (morning/afternoon), and the systematic use of oral carbohydrate drinks may add context to our findings [7].

## 5. Conclusions

This secondary analysis concludes that excessive preoperative liquid fasting is highly frequent in Romania, outlining the need for national and institutional strategies to overcome the differences between knowledge and practice. Apart from medical professionals, patients, and their families, management must be included in these initiatives, given the multifaceted aspects of prolonged fasting and guidelines non-adherence, including level of care, workload and emergency settings. Furthermore, future interventional studies could assess outcomes based on the two different fluid fasting times described—SIPS and true fluid fasting—as both patient comfort and preserved physiology are equally important.

## Figures and Tables

**Table 1:** Comparative analysis of workload, fluid fasting times and protocols across hospital categories.

	Hospital Category				
	Category Ia (n = 367, 16.8%)	Category Ib (n = 1096, 50.16%)	Category II (n = 499, 22.83%)	Category III (n = 223, 10.2%)	*p* Value
Theatres	13 [8 to 16]	27 [19 to 41]	19 [14 to 22]	10 [10 to 12]	<0.001
Anesthesiologists	8 [6 to 12]	17 [17 to 19]	14 [14 to 15]	6 [6 to 7]	<0.001
Procedures/year % Elective % Emergency	4993 [3048 to 6027] 86.64 [65 to 99.5] 13.35 [0.45 to 35]	18,750 [10,500 to 20,829] 79 [70 to 88.8] 21 [11.2 to 30]	9500 [6995 to 11,276] 71 [70 to 73.7] 29 [26.3 to 30]	1800 [1800 to 3358] 66.6 [66 to 77] 33.4 [22.2 to 33.4]	<0.001 <0.001 <0.001
Anesthesiologist/Theater	0.75 [0.61 to 0.75]	0.65 [0.41 to 0.85]	0.63 [0.63 to 1]	0.7 [0.5 to 0.7]	<0.001
Procedures/Theater	376.68 [376.68 to 384.1]	508.02 [508.02 to 610.05]	500 [499.6 to 512]	250 [150 to 335.8]	<0.001
Procedures/Anesthesiologist	502.25 [502.25 to 624.13]	986.84 [625 to 1225.24]	805.4 [499.6 to 805.4]	300 [300 to 479.7]	<0.001
Protocol NPO after midnight Fluids until morning Guidelines-based	104/367 (28.3%) 263/367 (71.7%) 0/367 (0%)	50/1096 (4.6%) 233/1096 (21.3%) 813/1096 (74.2%)	93/499 (18.6%) 194/499 (38.9%) 212/499 (42.5%)	173/223 (77.6%) 0/233 (0%) 50/233 (22.4%)	<0.001
SIPS time	8 h [3:50 to 12:00]	8 h [3:00 to 13:00]	10:20 h [4:00 to 14:00]	5 h [3:00 to 12:00]	
NOT SIPS time	12 h [9:30 to 14:20]	12 h [10:00 to 14:30]	13:30 h [11:00 to 15:30]	11 h [9:00 to 13:00]	<0.001

All variables except for protocol are continuous and are expressed as median and interquartile range. For protocols, the absolute and relative frequencies were reported. The Kruskal–Wallis test was used to compare the difference between hospital categories for continuous variables, while the Chi-square test was used to test the difference in protocol frequency between hospital categories. Abbreviations: NPO = *nil per os*.

**Table 2:** Binary logistic multivariable regression for prolonged true fluid fasting (NOT SIPS) prediction.

	B	Standard Error	*p* Value	OR	95% C.I. for OR	
					Lower	Upper
Hospital category Ia (reference)			0.044			
Hospital category Ib	0.481	0.466	0.302	1.618	0.649	4.037
Hospital category II	1.161	0.557	0.037	3.194	1.073	9.510
Hospital category III	−0.501	0.634	0.430	0.606	0.175	2.100
Protocol: NPO after midnight (reference)			0.117			
Protocol: Fluids allowed until morning	−0.376	0.473	0.427	0.687	0.272	1.736
Protocol: Guidelines-based	−1.027	0.521	0.049	0.358	0.129	0.994
Emergency procedures rate	0.055	0.012	<0.001	1.057	1.033	1.081
Afternoon schedule	0.528	0.647	0.415	1.695	0.477	6.028
Sex	−0.325	0.203	0.110	0.723	0.485	1.077
Constant	2.324	0.475	<0.001	10.218		

Abbreviations: C.I. = confidence interval; NPO = nil per os, OR = odds ratio.

**Table 3:** Binary logistic multivariable regression for prolonged SIPS time prediction.

	B	Standard Error	*p* Value	OR	95% C.I. for OR	
					Lower	Upper
Hospital category Ia (reference)			<0.001			
Hospital category Ib	−0.025	0.171	0.885	0.976	0.698	1.363
Hospital category II	0.079	0.181	0.665	1.082	0.758	1.544
Hospital category III	−1.401	0.246	<0.001	0.246	0.152	0.399
Protocol: NPO after midnight (reference)			<0.001			
Protocol: Fluids allowed until morning	−0.871	0.179	<0.001	0.419	0.295	0.595
Protocol: Guidelines-based	−0.951	0.179	<0.001	0.386	0.272	0.549
Emergency procedures rate	0.010	0.005	0.047	1.010	1.000	1.019
Afternoon schedule	0.045	0.191	0.814	1.046	0.720	1.520
Sex	0.092	0.095	0.332	1.096	0.910	1.320
Constant	1.373	0.189	<0.001	3.946		

Abbreviations: C.I. = confidence interval; NPO = nil per os, OR = odds ratio.

## Data Availability

The data presented in this study are available on reasonable request from the corresponding author.

## Supplementary Materials

The following supporting information can be downloaded at: https://www.mdpi.com/article/10.3390/nu18111714/s1, File S1: Hospital category and workload indices reported by participating centres from Romania; File S2: STROBE Statement—Checklist of items that should be included in reports of cohort studies for the following study: True Pre-operative Liquid Fasting in Romania—A Secondary Analysis of the Thirst Study; File S3: Median SIPS and true fluid fasting (NOT SIPS) times with heatmaps of all the pairwise comparisons in average rank difference across centres, regions and procedures; File S4. Mixed models with fixed and random effects for estimation of workload indices on fluid fasting time. Scatterplot exploratory visualization of workload indices distribution compared to fluid fasting time. File S5: Linear Mixed Models for SIPS and true fluid fasting (NOT SIPS times); File S6. Liberal (<2 h), 2-4 h and prolonged (>4 h) rates for SIPS and true fluid fasting (NOT SIPS) times across centres, procedures, hospital category and protocol in place.

## Abbreviations

The following abbreviations are used in this manuscript:

ASA	American Society of Anesthesiologists
Cap	Capital
Cen	Central
CI	Confidence Interval
ENT	Ear–Nose–Throat
EQUATOR	Enhancing the QUAlity and Transparency Of health Research
ESAIC	European Society of Anaesthesiology and Intensive Care
NE	Northeast
NORA	Non-Operating Room Anesthesia
NW	Northwest
OG	Obstetrics–Gynecology
SE	Southeast
SPSS	Statistical Package for Social Sciences
STROBE	Strengthening the Reporting of Observational Studies in Epidemiology
SW	Southwest
W	West
