# Supplementary material for: True Preoperative Liquid Fasting in Romania—A Secondary Analysis of the Thirst Study
Source: Nutrients. 2026 May 27;18(11):1714. doi: 10.3390/nu18111714 (PMC13259189; doi:10.3390/nu18111714)
Supplement: Supplementary file 1 [file nutrients-18-01714-s001.zip › Supplementary material S1.pdf]

Supplementary Material File S1: Hospital category and workload indices reported by participating centres from Romania

Table S1.1. Hospital category and workload indices

Centre	Hospital category	Number of theatres	Number of anesthesiologists	Number of procedures/year	% Elective	% Emergency	Anesthesiologist-to-theatre	Procedures-to-theatre	Proceudres-to-anesthesiologist
3 (n=54)	1a	8	6	917	80.37	19.63	0.75	114.63	152.83
4 (n=50)	1a	5	5	3048	86.65	13.35	1	609.6	609.6
8 (n=159)	1a	16	12	6027	99.55	0.45	0.75	376.69	502.25
10 (n=104)	1a	13	8	4993	65.01	34.99	0.62	384.08	624.13
1 (n=481)	1b	41	17	20829	88.82	11.18	0.41	508.02	1225.2
2 (n=170)	1b	17	11	10371	79	21	0.65	610.06	942.82
5 (n=162)	1b	27	23	10500	66.67	33.33	0.85	388.89	456.52
6 (n=51)	1b	19	21	11863	69.96	30.05	1.11	624.37	564.9
11 (n=59)	1b	9	8	5000	70	30	0.89	555.56	625
12 (n=123)	1b	25	19	18750	70	30	0.76	750	986.84
21 (n=50)	1b	30	29	16343	78.92	21.08	0.97	544.77	563.55
7 (n=90)	2	19	17	9500	73.68	26.32	0.89	500	558.82
9 (n=93)	2	14	14	6995	67.65	2265	1	499.64	499.64

13 (n=72)	2	35	15	15000	70	30	0.43	428.57	1000
15 (n=160)	2	22	14	11276	71	29	0.64	512.55	805.43
18 (n=50)	2	8	5	4141	86.4	13.6	0.63	517.3	828.2
20 (n=34)	2	9	9	700	71.43	28.57	1	77.78	77.78
14 (n=78)	3	12	6	1800	77.78	22.22	0.5	150	300
16 (n=64)	3	10	7	3358	66.62	33.38	0.7	335.8	479.71
17 (n=50)	3	6	5	1500	45.13	54.87	0.83	250	300
19 (n=31)	3	14	7	2834	70.57	29.43	0.5	202.43	404.86
