# Supplementary material for: True Preoperative Liquid Fasting in Romania—A Secondary Analysis of the Thirst Study
Source: Nutrients. 2026 May 27;18(11):1714. doi: 10.3390/nu18111714 (PMC13259189; doi:10.3390/nu18111714)
Supplement: Supplementary file 1 [file nutrients-18-01714-s001.zip › Supplementary material S3.pdf]

Supplementary Material File S3– Median SIPS and true fluid fasting (NOT SIPS) times with heatmaps of all the pairwise comparisons in average rank difference across centres, regions and procedures

Table 3.1. SIPS and true fluid fasting (NOT SIPS) times across centres

	SIPS<br>Kruskal to Wallis test, p < 0.001		NOT SIPS<br>Kruskal to Wallis test, p < 0.001			
Centre	Median, [IQR]<br>h:m, [h:m to h:m]	Min to Max,<br>h:m to h:m	Median [IQR]<br>h:m, [h:m to h:m]	Min to Max<br>h:m to h:m	Time/rec. fasting time	Min to Max<br>h:m to h:m
All (*n*=2185)	8:00 [3:30 to 13:00]	0:10 to 36:45	12:00 [10:00 to 15:00]	0:30 to 36:45	6 [5 to 7.5]	0.25 to18.4
1 (*n*=481)	4:50 [2:30 to 12:00]	0:10 to 25:00	13:00 [10:00 to 15:00]	0:30 to 30:00	6.5 [5 to 7.5]	0.25 to 15
2 (*n*=170)	10:00 [5:00 to 13:22]	1:30 to 26:00	12:00 [10:00 to 14:00]	2:10 to 30:00	6 [5 to 7]	1.1 to 15
3 (*n*=54)	8:40 [5:34 to 11:38]	1:00 to 16:10	11:23 [9:00 to 13:23]	5:35 to 19:30	5.7 [4.5 to 6.7]	2.8 to 9.8
4 (*n*=50)	9:00 [6:00 to 12:00]	2:00 to 21:00	12:00 [10:00 to 14:00]	4:00 to 22:00	6 [5 to 7]	2 to 11
5 (*n*=162)	8:48 [3:34 to 12:00]	1:00 to 36:45	12:00 [9:30 to 14:00]	1:55 to 36:45	6 [4.75 to 7]	1 to 18.4
6 (*n*=51)	12:10 [4:20 to 15:10]	1:00 to 26:00	13:30 [11:20 to 16:00]	4:50 to 26:00	6.75 [5.66 to 8]	2.4 to 13
7 (*n*=90)	12:20 [9:08 to 15:23]	1:00 to 25:10	14:00 [11:10 to 16:20]	6:00 to 25:10	7 [3 to 12.6]	3 to 12.6
8 (*n*=159)	6:00 [3:30 to 12:00]	1:00 to 24:00	11:40 [7:00 to 13:30]	1:00 to 24:00	5.83 [3.5 to 6.75]	0.5 to 12
9 (*n*=93)	8:30 [4:00 to 12:20]	1:00 to 22:00	12:00 [9:15 to 14:30]	4:00 to 24:00	6 [4.63 to 7.25]	2 to 12
10 (*n*=104)	8:10 [3:00 to 14:00]	0:30 to 26:00	14:00 [10:49 to 16:00]	1:35 to 27:00	7 [5.4 to 8]	0.8 to 13.5
11 (*n*=59)	8:30 [3:00 to 12:15]	1:00 to 18:00	11:00 [9:15 to 13:00]	3:00 to 18:00	5.5 [4.63 to 6.5]	1.5 to 9
12 (*n*=123)	10:00 [6:00 to 13:00]	10:00 to 36:45	12:00 [10:00 to 15:00]	1:30 to 36:45	6 [5 to 7.5]	0.75 to 18.4
13 (*n*=72)	12:00 [3:30 to 14:28]	1:00 to 21:00	14:00 [12:00 to 15:00]	8:00 to 21:00	7 [6 to 7.5]	4 to 10.5
14 (*n*=78)	4:00 [3:00 to 8:26]	1:00 to 16:00	10:30 [8:11 to 13:00]	2:00 to 16:00	5.25 [4.1 to 6.5]	1 to 8
15 (*n*=160)	5:00 [2:46 to 12:14]	1:00 to 27:00	14:00 [12:00 to 16:34]	2:50 to 36:45	7 [6 to 8.3]	1.4 to 18.4
16 (*n*=64)	12:00 [8:00 to 14:45]	1:30 to 24:00	13:00 [12:00 to 16:00]	4:00 to 24:00	6.5 [6 to 8]	2 to 12
17 (*n*=50)	2:05 [1:44 to 4:00]	1:00 to 12:00	9:15 [8:39 to 12:00]	4:00 to 15:00	4.63 [4.32 to 6]	2 to 7.5
18 (*n*=50)	12:00 [8:38 to 14:08]	2:00 to 24:00	12:00 [8:38 to 14:08]	2:00 to 24:00	6 [4.31 to 7.06]	1 to 12
19 (*n*=31)	6:00 [3:00 to 12:00]	2:00 to 16:00	12:00 [10:00 to 13:00]	3:00 to 16:00	6 [5 to 6.5]	1.5 to 8
20 (*n*=34)	10:00 [4:00 to 13:00]	2:00 to 24:00	12:30 [10:00 to 15:38]	6:35 to 24:00	6.25 [5 to 7.81]	3.3 to 12
21 (*n*=50)	12:00 [9:45 to 14:15]	3:00 to 35:45	12:00 [11:00 to 14:15]	4:00 to 36:45	6 [5.5 to 7.13]	2 to 18.4

Results expressed as median and IQR; Min=minimum; Max = maximum; time/rec. fasting time = actual median time divided by the recommended fasting time of 2 hours; Multiple comparisons across centres were performed and the *p* values have been adjusted by the Bonferroni correction.

Table 3.2. Heatmap with the pairwise comparisons of the average rank difference in SIPS time across centres using the Bonferroni correction.

	1	2	3	4	5	6	7	8	9	10	11	12	13	14	15	16	17	18	19	20	21
1		-316.3	-223.5	-287.6	-190.1	-414.2	-546.5	-93.4	-189.9	-218.2	-113.6	-321.3	-351.1	-113.9	-64.0	-504.8	-508.6	-506.3	-53.7	-314.5	-590.2
2	316.3		-92.8	-28.7	-126.2	-97.9	-230.2	-222.9	-126.4	-98.1	-202.8	-5.0	-34.8	-430.2	-252.3	-188.5	-824.9	-189.9	-262.6	-1.8	-273.9
3	223.5	92.8		-64.0	-33.4	-190.7	-322.9	-130.1	-33.6	-5.3	-110.0	-97.7	-127.6	-337.5	-159.6	-281.3	-732.2	-282.7	-169.8	-91.0	-366.7
4	287.6	28.7	64.0		-97.4	-126.7	-258.9	-194.1	-97.7	-69.3	-174.0	-33.7	-63.5	-401.5	-223.6	-217.2	-796.2	-218.7	-233.8	-26.9	-302.7
5	190.1	126.2	33.4	97.4		-224.1	-356.4	-96.7	-0.2	-28.1	-76.6	-131.2	-161.0	-304.1	-126.2	-314.7	-698.7	-316.1	-136.4	-124.4	-400.1
6	414.2	97.9	190.7	126.7	224.1		-132.3	-320.8	-224.3	-196.0	-300.7	-92.9	-63.1	-528.2	-350.3	-90.6	-922.8	-92.0	-360.5	-99.7	-176.0
7	546.5	230.2	322.9	258.9	356.4	132.3		-453.0	-356.6	-328.2	-432.9	-225.2	-195.4	-660.4	-482.5	-41.7	-1055.1	-40.2	-492.7	-232.0	-43.8
8	93.4	222.9	130.1	194.1	96.7	320.8	453.0		-96.5	-124.8	-20.1	-227.8	-257.7	-207.4	-29.5	-411.4	-602.1	-412.8	-39.7	-221.1	-496.8
9	189.9	126.4	33.6	97.7	0.2	224.3	356.6	96.5		-28.3	-76.3	-131.4	-161.2	-303.8	-125.9	-314.9	-698.5	-316.4	-136.2	-124.6	-400.3
10	218.2	98.1	5.3	69.3	28.1	196.0	328.2	124.8	28.3		-104.7	-103.0	-132.9	-332.2	-154.3	-286.6	-726.9	-288.0	-164.5	-96.3	-372.0
11	113.6	202.8	110.0	174.0	76.6	300.7	432.9	20.1	76.3	104.7		-207.7	-237.6	-227.5	-49.6	-391.3	-622.2	-392.7	-59.8	-201.0	-476.7
12	321.3	5.0	97.7	33.7	131.2	92.9	225.2	227.8	131.4	103.0	207.7		-29.8	-435.2	-257.3	-183.5	-829.9	-185.0	-267.6	-6.8	-268.9
13	351.1	34.8	127.6	63.5	161.0	63.1	195.4	257.7	161.2	132.9	237.6	29.8		-465.0	-287.1	-153.7	-859.7	-155.1	-297.4	-36.6	-239.1
14	113.9	430.2	337.5	401.5	304.1	528.2	660.4	207.4	303.8	332.2	227.5	435.2	465.0		-177.9	-618.7	-394.7	-620.2	-167.7	-428.4	-704.2
15	64.0	252.3	159.6	223.6	126.2	350.3	482.5	29.5	125.9	154.3	49.6	257.3	287.1	177.9		-440.9	-572.6	-442.3	-10.2	-250.6	-526.3
16	504.8	188.5	281.3	217.2	314.7	90.6	41.7	411.4	314.9	286.6	391.3	183.5	153.7	618.7	440.9		-1013.4	-1.4	-451.1	-190.3	-85.4
17	508.6	824.9	732.2	796.2	698.7	922.8	1055.1	602.1	698.5	726.9	622.2	829.9	859.7	394.7	572.6	1013.4		-1014.9	-562.4	-823.1	-1098.9
18	506.3	189.9	282.7	218.7	316.1	92.0	40.2	412.8	316.4	288.0	392.7	185.0	155.1	620.2	442.3	1.4	1014.9		-452.5	-191.7	-84.0
19	53.7	262.6	169.8	233.8	136.4	360.5	492.7	39.7	136.2	164.5	59.8	267.6	297.4	167.7	10.2	451.1	562.4	452.5		-260.8	-536.5
20	314.5	1.8	91.0	26.9	124.4	99.7	232.0	221.1	124.6	96.3	201.0	6.8	36.6	428.4	250.6	190.3	823.1	191.7	260.8		-275.7
21	590.2	273.9	366.7	302.7	400.1	176.0	43.8	496.8	400.3	372.0	476.7	268.9	239.1	704.2	526.3	85.4	1098.9	84.0	536.5	275.7	

Legend

**Blue**	The darker the blue, the more negative the difference between nodes; Adjusted p value < 0.05
**Orange**	The darker the orange, the more positive the rank difference between nodes; Adjusted p value < 0.05
**White**	Not significant – adjusted p value ≥ 0.05

Table 3.3. Heatmap with the pairwise comparisons of the average rank difference in true fluid fasting (NOT SIPS) time across centres using the Bonferroni correction.

Center	1	2	3	4	5	6	7	8	9	10	11	12	13	14	15	16	17	18	19	20	21
1		-73.0	-200.2	-57.5	-130.7	-198.7	-226.9	-241.9	-69.0	-179.5	-250.6	-0.4	-213.7	-373.4	-301.8	-187.7	-486.4	-134.8	-194.4	-42.3	-31.8
2	73.0		-127.2	-15.5	-57.7	-271.7	-299.9	-168.9	-4.0	-252.5	-177.6	-72.7	-286.7	-300.4	-374.8	-260.7	-413.4	-61.8	-121.4	-115.3	-104.8
3	200.2	127.2		-142.7	-69.5	-398.9	-427.1	-41.7	-131.3	-379.7	-50.4	-199.9	-413.9	-173.2	-502.0	-388.0	-286.2	-65.4	-5.9	-242.5	-232.1
4	57.5	15.5	142.7		-73.2	-256.2	-284.4	-184.4	-11.5	-237.0	-193.1	-57.2	-271.2	-315.9	-359.3	-245.2	-428.9	-77.3	-136.9	-99.8	-89.3
5	130.7	57.7	69.5	73.2		-329.4	-357.6	-111.2	-61.8	-310.2	-119.9	-130.4	-344.4	-242.7	-432.5	-318.5	-355.7	-4.1	-63.6	-173.0	-162.5
6	198.7	271.7	398.9	256.2	329.4		-28.2	-440.6	-267.7	-19.2	-449.3	-199.0	-15.0	-572.1	-103.1	-11.0	-685.1	-333.5	-393.1	-156.4	-166.9
7	226.9	299.9	427.1	284.4	357.6	28.2		-468.8	-295.9	-47.4	-477.5	-227.3	-13.2	-600.3	-74.9	-39.2	-713.3	-361.7	-421.3	-184.6	-195.1
8	241.9	168.9	41.7	184.4	111.2	440.6	468.8		-172.9	-421.4	-8.7	-241.6	-455.6	-131.5	-543.7	-429.6	-244.5	-107.1	-47.5	-284.2	-273.7
9	69.0	4.0	131.3	11.5	61.8	267.7	295.9	172.9		-248.4	-181.6	-68.6	-282.7	-304.5	-370.8	-256.7	-417.4	-65.8	-125.4	-111.3	-100.8
10	179.5	252.5	379.7	237.0	310.2	19.2	47.4	421.4	248.4		-430.1	-179.8	-34.2	-552.9	-122.3	-8.3	-665.9	-314.3	-373.8	-137.2	-147.7
11	250.6	177.6	50.4	193.1	119.9	449.3	477.5	8.7	181.6	430.1		-250.3	-464.3	-122.8	-552.4	-438.3	-235.8	-115.8	-56.2	-292.9	-282.4
12	0.4	72.7	199.9	57.2	130.4	199.0	227.3	241.6	68.6	179.8	250.3		-214.1	-373.1	-302.2	-188.1	-486.1	-134.4	-194.0	-42.6	-32.2
13	213.7	286.7	413.9	271.2	344.4	15.0	13.2	455.6	282.7	34.2	464.3	214.1		-587.1	-88.1	-26.0	-700.1	-348.5	-408.1	-171.4	-181.9
14	373.4	300.4	173.2	315.9	242.7	572.1	600.3	131.5	304.5	552.9	122.8	373.1	587.1		-675.2	-561.2	-113.0	-238.6	-179.1	-415.7	-405.2
15	301.8	374.8	502.0	359.3	432.5	103.1	74.9	543.7	370.8	122.3	552.4	302.2	88.1	675.2		-114.1	-788.2	-436.6	-496.2	-259.5	-270.0
16	187.7	260.7	388.0	245.2	318.5	11.0	39.2	429.6	256.7	8.3	438.3	188.1	26.0	561.2	114.1		-674.1	-322.5	-382.1	-145.4	-155.9
17	486.4	413.4	286.2	428.9	355.7	685.1	713.3	244.5	417.4	665.9	235.8	486.1	700.1	113.0	788.2	674.1		-351.6	-292.0	-528.7	-518.2
18	134.8	61.8	65.4	77.3	4.1	333.5	361.7	107.1	65.8	314.3	115.8	134.4	348.5	238.6	436.6	322.5	351.6		-59.6	-177.1	-166.6
19	194.4	121.4	5.9	136.9	63.6	393.1	421.3	47.5	125.4	373.8	56.2	194.0	408.1	179.1	496.2	382.1	292.0	59.6		-236.7	-226.2
20	42.3	115.3	242.5	99.8	173.0	156.4	184.6	284.2	111.3	137.2	292.9	42.6	171.4	415.7	259.5	145.4	528.7	177.1	236.7		-10.5
21	31.8	104.8	232.1	89.3	162.5	166.9	195.1	273.7	100.8	147.7	282.4	32.2	181.9	405.2	270.0	155.9	518.2	166.6	226.2	10.5	

Legend

**Blue**	The darker the blue, the more negative the difference between nodes; Adjusted p value < 0.05
**Orange**	The darker the orange, the more positive the rank difference between nodes; Adjusted p value < 0.05
**White**	Not significant – adjusted p value ≥ 0.05

Table 3.4. SIPS and true fluid fasting (NOT SIPS) times across procedures

	SIPS<br>Kruskal to Wallis test, p < 0.001		NOT SIPS<br>Kruskal to Wallis test, p < 0.001	
Procedure (*n*=)	Median [IQR]<br>h:m, [h:m to h:m]	Min to Max<br>h:m to h:m	Median [IQR]<br>h:m, [h:m to h:m]	Min to Max<br>h:m to h:m
All (*n*=2185)	8:00 [3:30 to 13:00]	0:10 to 36:45	12:00 [10:00 to 15:00]	0:30 to 36:45
Cardiothoracic (*n*=34)	11:00 [3:00 to 14:00]	2:00 to 19:00	12:05 [11:00 to 14:00]	2:00 to 19:00
Endoscopy (*n*=427)	8:45 [4:00 to 13:00]	1:00 to 36:45	12:00 [10:00 to 14:00]	1:35 to 36:45
ENT (*n*=78)	9:30 [4:00 to 13:05]	0:30 to 21:00	12:00 [10:00 to 14:00]	1:30 to 21:00
General surgery (*n*=624)	10:00 [4:00 to 13:43]	0:30 to 36:45	12:25 [10:00 to 15:00]	1:00 to 36:45
Neurosurgery (*n*=72)	8:00 [5:00 to 12:00]	1:00 to 25:00	12:00 [10:00 to 14:45]	4:50 to 25:00
NORA (*n*=43)	10:30 [4:30 to 16:15]	2:00 to 24:00	14:00 [11:00 to 18:00]	3:00 to 36:45
Obstetrics to Gynaecology (*n*=225)	6:00 [3:00 to 12:00]	1:00 to 26:00	12:00 [10:08 to 14:40]	1:00 to 30:00
Ophthalmology (*n*=139)	2:30 [1:00 to 4:00]	0:10 to 22:00	12:00 [4:00 to 15:00]	0:30 to 22:00
Orthopaedic surgery (*n*=250)	9:00 [3:00 to 13:00]	0:30 to 26:00	12:05 [10:00 to 14:45]	2:00 to 30:00
Other (*n*=36)	8:20 [3:15 to 13:08]	1:00 to 24:00	12:10 [8:20 to 14:00]	2:00 to 24:00
Plastic (*n*=46)	4:00 [2:00 to 10:00]	1:00 to 16:45	12:00 [9:00 to 13:15]	1:55 to 22:00
Urology (*n*=168)	8:00 [3:30 to 13:15]	1:00 to 27:00	12:30 [10:55 to 15:30]	2:50 to 27:00
Vascular surgery (*n*=43)	10:00 [3:30 to 13:35]	1:00 to 18:00	12:55 [10:00 to 15:00]	3:30 to 18:00

Results expressed as median and IQR; Mi n=minimum; Max = maximum;
Multiple comparisons across centres were performed and *p* values have been adjusted by the Bonferroni correction. This analysis is available in Supplementary material x.

Table 3.5. Heatmap with the pairwise comparisons of the average rank difference in SIPS time across procedures using the Bonferroni correction.

	Ophtalmology	Plastic	OG	Orthopaedic	Other	Urology	Neurosurgery	Vascular	ENT	Cardiothoracic	Endoscopy	General	NORA
**Ophtalmology**		-317.0	530.2	-621.8	-637.6	-650.6	654.7	-660.6	669.7	671.9	687.2	719.9	849.3
**Plastic**	317.0		213.1	304.8	320.6	-333.6	337.6	-343.6	352.6	354.8	370.2	402.9	532.2
**OG**	-530.2	-213.1		-91.7	-107.5	-120.4	124.5	-130.5	139.5	141.7	157.1	189.8	319.1
**Orthopaedic**	621.8	-304.8	91.7		-15.8	-28.8	32.9	-38.8	47.8	50.0	65.4	98.1	227.5
**Other**	637.6	-320.6	107.5	15.8		-12.9	17.0	-23.0	32.0	34.2	49.6	82.3	211.6
**Urology**	650.6	333.6	120.4	28.8	12.9		4.1	-10.0	19.1	21.3	36.6	69.3	198.7
**Neurosurgery**	-654.7	-337.6	-124.5	-32.9	-17.0	-4.1		-5.9	15.0	17.2	32.6	65.3	-194.6
**Vascular**	660.6	343.6	130.5	38.8	23.0	10.0	5.9		9.0	11.2	26.6	59.3	188.7
**ENT**	-669.7	-352.6	-139.5	-47.8	-32.0	-19.1	-15.0	-9.0		2.2	17.6	-50.3	-179.6
**Cardiothoracic**	-671.9	-354.8	-141.7	-50.0	-34.2	-21.3	-17.2	-11.2	-2.2		-15.4	-48.1	-177.4
**Endoscopy**	-687.2	-370.2	-157.1	-65.4	-49.6	-36.6	-32.6	-26.6	-17.6	15.4		-32.7	-162.0
**General**	-719.9	-402.9	-189.8	-98.1	-82.3	-69.3	-65.3	-59.3	50.3	48.1	32.7		-129.3
**NORA**	-849.3	-532.2	-319.1	-227.5	-211.6	-198.7	194.6	-188.7	179.6	177.4	162.0	129.3	

Legend

**Blue**	The darker the blue, the more negative the difference between nodes; Adjusted p value < 0.05
**Orange**	The darker the orange, the more positive the rank difference between nodes; Adjusted p value < 0.05
**White**	Not significant – adjusted p value ≥ 0.05

Table 3.6. Heatmap with the pairwise comparisons of the average rank difference in true fluid fasting (NOT SIPS) time across procedures using the Bonferroni correction.

	Ophtalmology	Plastic	Other	ENT	Endoscopy	Neurosurgery	Cardiothoracic	OG	Orthopaedic	Vascular	General	Urology	NORA
**Ophtalmology**		-20.1	-71.4	92.6	123.5	140.2	191.3	196.9	-207.4	-210.1	232.3	-281.4	419.6
**Plastic**	20.1		51.3	72.5	103.4	120.1	171.2	176.8	187.3	-190.0	212.2	-261.3	399.5
**Other**	71.4	-51.3		21.2	52.0	68.8	119.9	125.5	136.0	-138.7	160.9	-210.0	348.2
**ENT**	-92.6	-72.5	-21.2		30.8	-47.6	98.7	-104.3	-114.8	-117.5	-139.7	-188.8	-327.0
**Endoscopy**	-123.5	-103.4	-52.0	-30.8		-16.8	67.8	-73.5	-83.9	-86.6	-108.8	-158.0	-296.2
**Neurosurgery**	-140.2	-120.1	-68.8	47.6	16.8		51.1	-56.7	-67.1	-69.9	92.1	-141.2	-279.4
**Cardiothoracic**	-191.3	-171.2	-119.9	-98.7	-67.8	-51.1		-5.6	-16.1	-18.8	-41.0	-90.2	-228.4
**OG**	-196.9	-176.8	-125.5	104.3	73.5	56.7	5.6		-10.5	-13.2	35.4	-84.5	222.7
**Orthopaedic**	207.4	-187.3	-136.0	114.8	83.9	67.1	16.1	10.5		-2.7	24.9	-74.1	212.3
**Vascular**	210.1	190.0	138.7	117.5	86.6	69.9	18.8	13.2	2.7		22.2	71.4	209.6
**General**	-232.3	-212.2	-160.9	139.7	108.8	-92.1	41.0	-35.4	-24.9	-22.2		-49.1	-187.4
**Urology**	281.4	261.3	210.0	188.8	158.0	141.2	90.2	84.5	74.1	-71.4	49.1		138.2
**NORA**	-419.6	-399.5	-348.2	327.0	296.2	279.4	228.4	-222.7	-212.3	-209.6	187.4	-138.2	

Legend

**Blue**	The darker the blue, the more negative the difference between nodes; Adjusted p value < 0.05
**Orange**	The darker the orange, the more positive the rank difference between nodes; Adjusted p value < 0.05
**White**	Not significant – adjusted p value ≥ 0.05
