# Supplementary material for: True Preoperative Liquid Fasting in Romania—A Secondary Analysis of the Thirst Study
Source: Nutrients. 2026 May 27;18(11):1714. doi: 10.3390/nu18111714 (PMC13259189; doi:10.3390/nu18111714)
Supplement: Supplementary file 1 [file nutrients-18-01714-s001.zip › Supplementary material S4.pdf]

Supplementary Material File S4. Mixed models with fixed and random effects for estimation of workload indices on fluid fasting time. Scatterplot exploratory visualization of workload indices distribution compared to fluid fasting time.

Table S4.1 Mixed model 1 for estimation of effect size on NOT SIPS time

Parameter	Estimate	Standard Error	t	P value	95% Confidence Interval	
					Lower Bound	Upper Bound
Intercept	11.491	3.293	3.489	0.046	0.404	22.579
Hospital Category Ia	2.798	1.397	2.004	0.045	0.059	5.537
Hospital Category Ib	5.198	1.217	4.271	<0.001	2.811	7.584
Hospital Category II	4.011	.758	5.290	<0.001	2.524	5.498
Hospital Category III	0[b]	0	.	.	.	.
Protocol NPO after midnight	-1.926	.636	-3.029	0.002	-3.173	-0.679
Protocol Fluids until morning	-2.733	.929	-2.942	0.003	-4.555	-0.911
Protocol guidelines based (reference)						
Afternoon schedule = No	-1.513	1.096	-1.380	0.168	-3.663	0.637
Afternon schedule = Yes Reference						
Anesth-to-th Ratio = 0.41	-3.113	1.072	-2.903	0.004	-5.216	-1.010
Anesth-to-th Ratio = 0.43	-0.409	.680	-0.601	0.548	-1.742	0.924
Anesth-to-th Ratio = 0.5	2.426	.972	2.495	0.013	0.519	4.332
Anesth-to-th Ratio = 0.62	3.546	1.770	2.004	0.045	0.075	7.017

Anesth-to-th Ratio = 0.63	-2.530	.758	-3.337	<0.001	-4.018	-1.043
Anesth-to-th Ratio = 0.64	3.349	.879	3.809	<0.001	1.625	5.073
Anesth-to-th Ratio = 0.65	-3.081	1.105	-2.789	0.005	-5.247	-0.915
Anesth-to-th Ratio = 0.7	5.706	1.031	5.536	<0.001	3.685	7.727
Anesth-to-th Ratio = 0.75	0.518	1.612	0.321	0.748	-2.644	3.680
Anesth-to-th Ratio = 0.76	0.294	1.310	0.225	0.822	-2.275	2.864
Anesth-to-th Ratio = 0.83	0[b]	0	.	.	.	.
Anesth-to-th Ratio = 0.85	-3.327	1.107	-3.005	0.003	-5.498	-1.156
Anesth-to-th Ratio = 0.88	-2.999	.822	-3.648	<0.001	-4.611	-1.387
Anesth-to-th Ratio = 0.89	0[b]	0	.	.	.	.
Anesth-to-th Ratio = 0.97	0.094	1.214	0.077	0.939	-2.288	2.475
Anesth-to-th Ratio = 1	0[b]	0	.	.	.	.
Anesth-to-th Ratio = 1.1	0[b]	0	.	.	.	.
Sex = Female	0.075	.189	0.396	0.692	-0.295	0.445
Sex = Male	0[b]	0	.	.	.	.

a. Dependent Variable: NOT SIPS (median hours)

b. This parameter is set to zero because it is redundant.

Abbreviations: Anesth-to-th ratio = number of anesthesiologists/number of theatres; NPO = nil per os,

Table S4.2 Mixed model 2 for estimation of effect size on NOT SIPS time

					95% Confidence Interval	
Parameter	Estimate	Std. Error	t	Sig.	Lower Bound	Upper Bound
Intercept	12.054	3.052	3.950	.058	-.970	25.078
Sex = Female	.092	.189	.486	.627	-.278	.462
Sex = Male	0[b]	0	.	.	.	.
Proced-to-anesth=77.77	.707	.763	.927	.354	-.789	2.202
Proced-to-anest=152.83	-.694	.617	-1.125	.261	-1.903	.516
Proced-to-anest=300	-1.986	.429	-4.631	<.001	-2.827	-1.145
Proced-to-anest=404.86	-.746	.796	-.937	.349	-2.308	.815
Proced-to-anest=456.52	-.213	.390	-.546	.585	-.979	.553
Proced-to-anest=479.71	1.696	.572	2.966	.003	.575	2.817
Proced-to-anest=499.64	.001	.487	.003	.998	-.953	.956
Proced-to-anest=502.25	-1.505	.395	-3.806	<.001	-2.280	-.729
Proced-to-anest=558.82	1.926	.494	3.902	<.001	.958	2.894
Proced-to-anest=563.55	1.283	.639	2.008	.045	.030	2.535
Proced-to-anest=564.9	1.893	.633	2.991	.003	.652	3.134
Proced-to-anest=609.6	.299	.639	.469	.639	-.953	1.552
Proced-to-anest=624.13	1.527	.465	3.286	.001	.616	2.438

Proced-to-anest=625.	-1.107	.593	-1.866	.062	-2.269	.056
Proced-to-anest=805.43	2.541	.393	6.474	<.001	1.772	3.311
Proced-to-anest=828.2	-.606	.639	-.948	.343	-1.858	.647
Proced-to-anest=942.82	.030	.385	.077	.938	-.725	.784
Proced-to-anest=986.84	.676	.434	1.555	.120	-.176	1.528
Proced-to-anest=1000	1.517	.543	2.792	.005	.451	2.582
Proced-to-anest=1225.24	0[b]	0	.	.	.	.
Hospital Category I	0[b]	0	.	.	.	.
Hospital Category II	0[b]	0	.	.	.	.
Hospital Category III	0[b]	0	.	.	.	.
Hospital Category IV	0[b]	0	.	.	.	.
Afternoon schedule = No	0[b]	0	.	.	.	.
Afternon schedule = Yes	0[b]	0	.	.	.	.
Protocol NPO after midnight	0[b]	0	.	.	.	.
Protocol Fluids until morning	0[b]	0	.	.	.	.
Protocol guidelines based (reference)	0[b]	0	.	.	.	.

a. Dependent Variable: NOT SIPS (median hours)

b. This parameter is set to zero because it is redundant.

Abbreviations: NPO = nil per os, Proced-to-anest = procedures-to-anesthesiologist

Table S4.3 Mixed model 3 for estimation of effect size on NOT SIPS time

Parameter	Estimate	Std. Error	t	Sig.	95% Confidence Interval	
					Lower Bound	Upper Bound
Intercept	14.968	3.178	4.711	.030	3.121	26.815
Sex = Female	.089	.189	.473	.637	-.281	.460
Sex = Male	0^b	0	.	.	.	.
Hospital Category I	-2.614	1.082	-2.416	.016	-4.735	-.492
Hospital Category II	-1.020	.663	-1.540	.124	-2.320	.279
Hospital Category III	-2.301	.812	-2.836	.005	-3.893	-.710
Hospital Category IV	0^b	0	.	.	.	.
Afternoon schedule = No	-1.217	.716	-1.700	.089	-2.622	.187
Afternon schedule = Yes	0^b	0	.	.	.	.
Proced-to-th=77.77	.095	1.195	.079	.937	-2.248	2.437
Proced-to-th=114.63	.224	1.106	.202	.840	-1.946	2.393
Proced-to-th=150	-3.618	.727	-4.976	<.001	-5.044	-2.192
Proced-to-th=202.43	-2.442	.941	-2.596	.009	-4.287	-.598
Proced-to-th=250	-3.781	.811	-4.660	<.001	-5.372	-2.190
Proced-to-th=335.8	0^b	0	.	.	.	.
Proced-to-th=376.69	-.587	1.000	-.587	.557	-2.548	1.374
Proced-to-th=384.1	2.444	1.029	2.375	.018	.426	4.463

Proced-to-th=388.88	-.889	.514	-1.729	.084	-1.897	.120
Proced-to-th=428.57	2.122	.791	2.682	.007	.570	3.674
Proced-to-th=499.64	.607	.754	.804	.421	-.872	2.085
Proced-to-th=500	2.531	.758	3.339	<.001	1.044	4.018
Proced-to-th=508.02	-.675	.435	-1.555	.120	-1.528	.177
Proced-to-th=512.54	3.147	.696	4.519	<.001	1.781	4.512
Proced-to-th=517.62	0[b]	0	.	.	.	.
Proced-to-th=544.76	.607	.721	.842	.400	-.807	2.021
Proced-to-th=555.55	-2.999	.822	-3.649	<.001	-4.611	-1.388
Proced-to-th=609.6	0[b]	0	.	.	.	.
Proced-to-th=610.05	-.645	.511	-1.263	.207	-1.647	.356
Proced-to-th=624.37	0[b]	0	.	.	.	.
Proced-to-th=750	0[b]	0	.	.	.	.
Protocol NPO after midnight	0[b]	0	.	.	.	.
Protocol Fluids until morning	0[b]	0	.	.	.	.
Protocol guidelines based (reference)	0[b]	0	.	.	.	.

a. Dependent Variable: NOT SIPS (median hours)

b. This parameter is set to zero because it is redundant.

Abbreviations: Abbreviations: NPO = nil per os, Proced-to-th = procedures-to-theatres

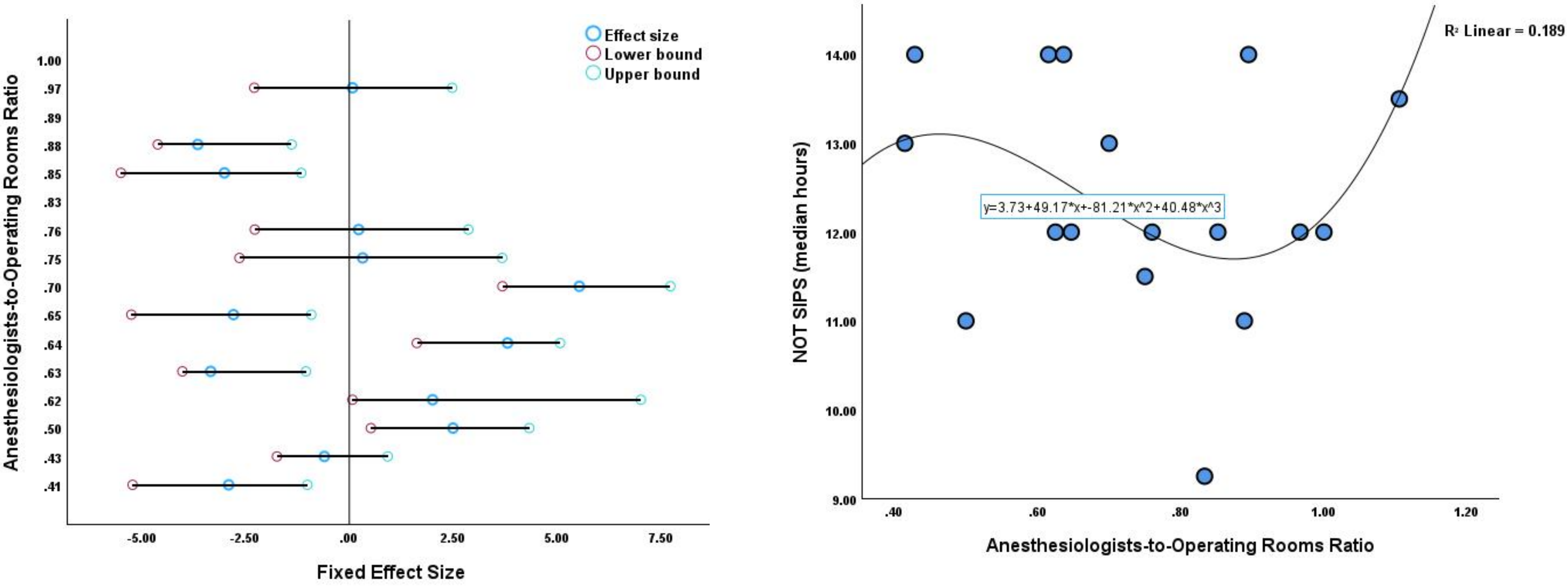

Figure S4.1 Forest plot of the estimates of fixed effect size for anesthesiologist-to-theatres ratio (left) and scatter plot representing the anesthesiologist-to-theatres ratio and median fluid fasting times (right). A weak possible cubic distribution can be observed in the right image, and this explains why patients with relatively unfavorable anesthesiologist-to-theatres ratio have totally opposite fluid fasting times.

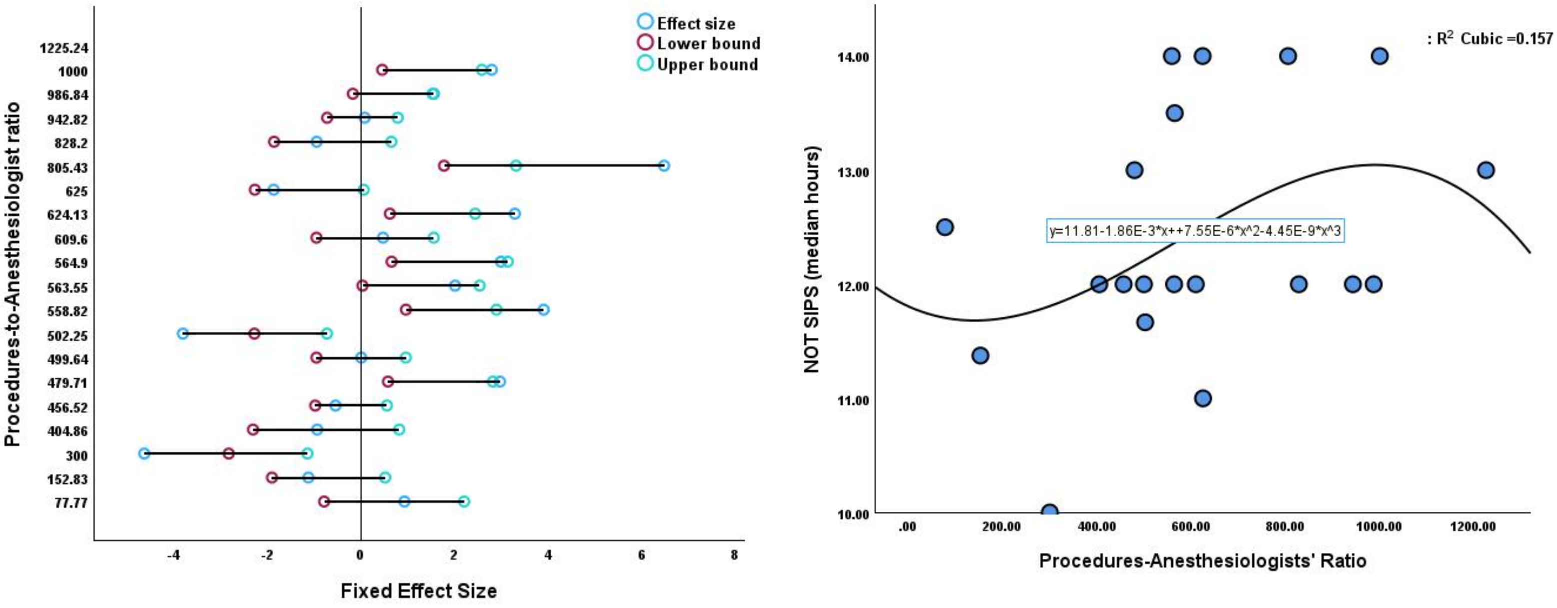

Figure S4.2 Forest plot of the estimates of fixed effect size for procedures-to-anesthesiologist ratio (left) and scatter plot representing the procedures-to-anesthesiologist ratio and median fluid fasting times (right). A weak possible cubic distribution can be observed in the right image, and this explains why patients with relatively unfavorable ratio have totally opposite fluid fasting times.

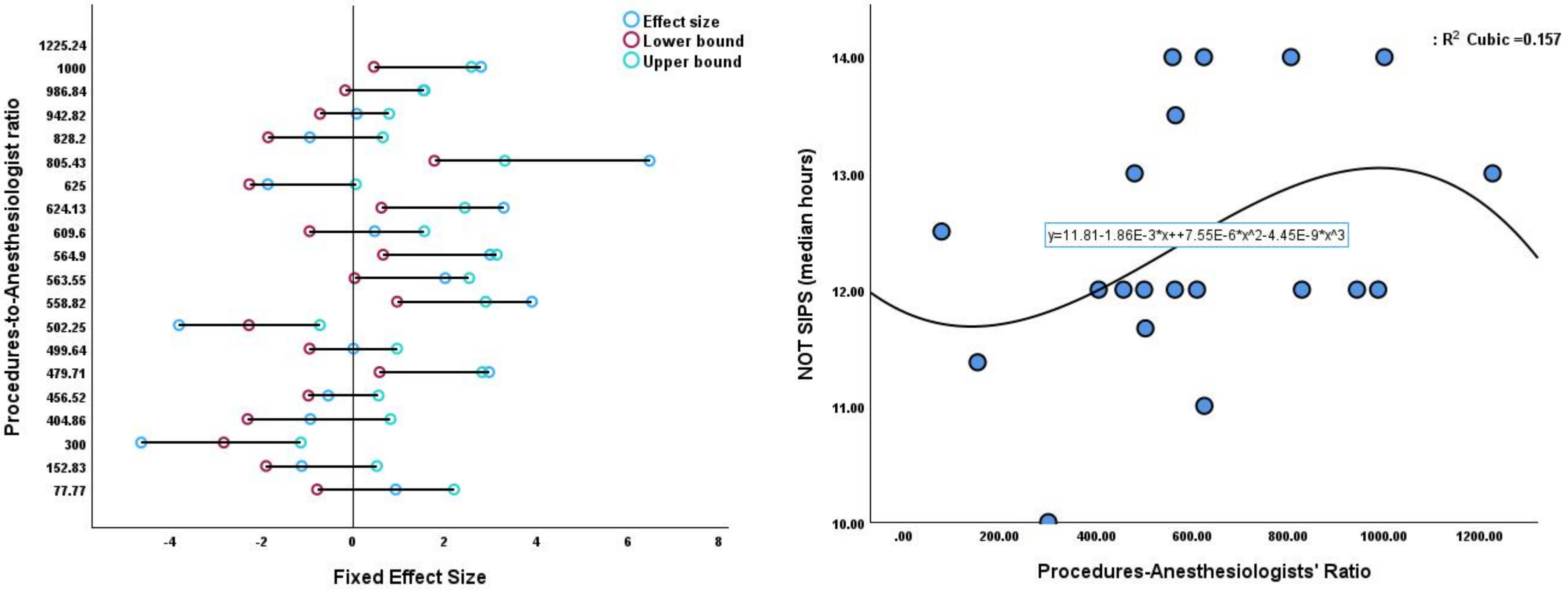

Figure S4.3 Forest plot of the estimates of fixed effect size for procedures-to-theatre ratio (left) and scatter plot representing the procedures-to-theatre ratio and median fluid fasting times (right). A weak possible cubic distribution can be observed in the right image, and this explains why patients with relatively unfavorable ratio have totally opposite fluid fasting times.
