# Supplementary material for: True Preoperative Liquid Fasting in Romania—A Secondary Analysis of the Thirst Study
Source: Nutrients. 2026 May 27;18(11):1714. doi: 10.3390/nu18111714 (PMC13259189; doi:10.3390/nu18111714)
Supplement: Supplementary file 1 [file nutrients-18-01714-s001.zip › Supplementary material S5.pdf]

Supplementary Material File S5: Linear Mixed Models for SIPS and true fluid fasting (NOT SIPS times)

Linear Mixed Models for SIPS

Model 1: Dependent: SIPS time, Independent Fixed Effect: Procedure, Independent Random Effect: Centre

Model dimension

**Information Criteria**	
-2 Restricted Log Likelihood	13468.59
Akaike's Information Criterion (AIC)	13472.59
Schwarz's Bayesian Criterion (BIC)	13483.95
**Coefficients of Determination**	
Pseudo-R Square	Marginal: 0.057
	Conditional: 0.176
**Intraclass Correlation Coefficients (ICC)**	
Overall ICCs	Adjusted: 0.126
	Conditional: 0.119

**Estimates of Fixed Effects**[a]							
Type III Tests for Fixed Effect: Procedure F = 9.857, p < 0.001							
						95% Confidence Interval	
Parameter	Estimate	Std. Error	df	t	Sig.	Lower Bound	Upper Bound
Intercept (Vascular)	8.732	.931	300.343	9.377	<.001	6.899	10.565

Cardiothoracic	.526	1.234	2163.791	.426	.670	-1.894	2.947
Endoscopy	.557	.874	2171.628	.638	.524	-1.156	2.271
ENT	.210	1.017	2162.770	.207	.836	-1.784	2.204
General Surgery	.634	.845	2165.848	.750	.454	-1.024	2.291
Neurosurgery	-.018	1.029	2162.378	-.018	.986	-2.037	2.000
NORA	3.063	1.167	2167.702	2.626	.009	.775	5.351
OG	-.427	.902	2169.328	-.474	.636	-2.195	1.341
Ophtalmology	-4.716	.970	2168.347	-4.861	<.001	-6.619	-2.814
Orthopaedic	-.233	.892	2167.419	-.261	.794	-1.982	1.516
Other	-.235	1.231	2172	-.191	.849	-2.648	2.179
Plastic	-2.055	1.150	2170.081	-1.787	.074	-4.311	.200
Urology	.270	.928	2172	.291	.771	-1.549	2.090

Model 2 Dependent: SIPS time, Independent Fixed Effect: Procedure, Independent Random Effect: Region

Model dimension

Information Criteria

-2 Restricted Log Likelihood	13571.91
Akaike's Information Criterion (AIC)	13575.91
Schwarz's Bayesian Criterion (BIC)	13587.27
**Coefficients of Determination**	
Pseudo-R Square	Marginal: 0.072
	Conditional: 0.111
**Intraclass Correlation Coefficients (ICC)**	
Overall ICCs	Adjusted: 0.042
	Conditional: 0.039

Estimates of Fixed Effects[a]

Type III Tests for Fixed Effect: Procedure F = 13.403, p < 0.001

Parameter	Estimate	Std. Error	df	t	Sig.	95% Confidence Interval Lower Bound	95% Confidence Interval Upper Bound
Intercept (Vascular)	9.058	.939	85.670	9.648	<.001	7.192	10.925
Cardiothoracic	-.193	1.252	2169.164	-.154	.877	-2.649	2.263
Endoscopy	.076	.875	2171.743	.087	.931	-1.639	1.791
ENT	-.204	1.039	2170.942	-.197	.844	-2.241	1.833

General Surgery	.457	.858	2169.889	.533	.594	-1.226	2.140
Neurosurgery	-.481	1.052	2169.047	-.457	.648	-2.544	1.582
NORA	1.358	1.178	2171.560	1.153	.249	-.952	3.668
OG	-1.335	.908	2171.380	-1.471	.141	-3.115	.445
Ophtalmology	-5.718	.967	2171.973	-5.915	<.001	-7.614	-3.823
Orthopaedic	-.612	.900	2170.927	-.680	.497	-2.376	1.153
Other	-.354	1.236	2171.861	-.286	.775	-2.777	2.070
Plastic	-3.120	1.155	2169.579	-2.701	.007	-5.385	-.855
Urology	-.008	.929	2169.342	-.009	.993	-1.831	1.814

Linear Mixed Models for NOT SIPS time

Model 1: Dependent: NOT SIPS time, Independent Fixed Effect: Procedure, Independent Random Effect: Centre

Model dimension

**Information Criteria**	
-2 Restricted Log Likelihood	12543.87
Akaike's Information Criterion (AIC)	12547.87
Schwarz's Bayesian Criterion (BIC)	12559.24
**Coefficients of Determination**	
Pseudo-R Square	Marginal: 0.038
	Conditional: 0.112
**Intraclass Correlation Coefficients (ICC)**	
Overall ICCs	Adjusted: 0.077
	Conditional: 0.075

Estimates of Fixed Effects[a]

Type III Tests for Fixed Effect: Procedure F = 5.608, p < 0.001

						95% Confidence Interval	
Parameter	Estimate	Std. Error	df	t	Sig.	Lower Bound	Upper Bound
Intercept (Vascular)	11.809	.718	559.757	16.441	<.001	10.398	13.220

Cardiothoracic	-.278	.999	2168.890	-.278	.781	-2.236	1.681
Endoscopy	.451	.706	2169.318	.639	.523	-.934	1.836
ENT	-.678	.823	2167.812	-.823	.410	-2.291	.936
General Surgery	.850	.684	2170.572	1.243	.214	-.491	2.191
Neurosurgery	.001	.833	2167.397	.001	.999	-1.633	1.635
NORA	2.358	.944	2171.756	2.499	.013	.507	4.208
OG	.513	.729	2171.980	.704	.482	-.917	1.942
Ophtalmology	-2.553	.785	2171.984	-3.254	.001	-4.092	-1.014
Orthopaedic	.570	.721	2171.630	.790	.430	-.845	1.984
Other	.028	.994	2165.472	.028	.978	-1.922	1.977
Plastic	-.082	.930	2171.748	-.088	.930	-1.906	1.741
Urology	.822	.750	2165.058	1.097	.273	-.648	2.292

Model 2: Dependent: NOT SIPS time, Independent Fixed Effect: Procedure, Independent Random Effect: Region

Model dimension

**Information Criteria**	
-2 Restricted Log Likelihood	12583.98
Akaike's Information Criterion (AIC)	12587.98
Schwarz's Bayesian Criterion (BIC)	12559.35
**Coefficients of Determination**	
Pseudo-R Square	Marginal: 0.028
	Conditional: 0.082
**Intraclass Correlation Coefficients (ICC)**	
Overall ICCs	Adjusted: 0.055
	Conditional: 0.054

Estimates of Fixed Effects[a]

Type III Tests for Fixed Effect: Procedure F = 4.944, p < 0.001

						95% Confidence Interval	
Parameter	Estimate	Std. Error	df	t	Sig.	Lower Bound	Upper Bound
Intercept (Vascular)	12.388	.773	70.868	16.029	<.001	10.847	13.929
Cardiothoracic	-.270	.997	2168.882	-.271	.787	-2.226	1.686
Endoscopy	-.081	.697	2171.177	-.116	.908	-1.447	1.285

ENT	-.598	.827	2170.320	-.723	.470	-2.220	1.024
General Surgery	.474	.684	2169.474	.694	.488	-.866	1.814
Neurosurgery	-.147	.838	2168.788	-.175	.861	-1.789	1.496
NORA	1.724	.938	2170.934	1.838	.066	-.116	3.564
OG	-.108	.723	2170.770	-.149	.881	-1.526	1.310
Ophtalmology	-2.415	.770	2171.471	-3.136	.002	-3.925	-.905
Orthopaedic	.013	.717	2170.407	.018	.986	-1.393	1.418
Other	-.966	.984	2171.309	-.981	.326	-2.897	.964
Plastic	-1.075	.920	2169.211	-1.169	.243	-2.879	.729
Urology	.572	.740	2169.042	.772	.440	-.880	2.023
