# Supplementary material for: True Preoperative Liquid Fasting in Romania—A Secondary Analysis of the Thirst Study
Source: Nutrients. 2026 May 27;18(11):1714. doi: 10.3390/nu18111714 (PMC13259189; doi:10.3390/nu18111714)
Supplement: Supplementary file 1 [file nutrients-18-01714-s001.zip › Supplementary material S6.pdf]

Supplementary Material File S6. Liberal (<2 hours), 2-4 hours and prolonged (>4 hours) rates for SIPS and true fluid fasting (NOT SIPS) times across centres, procedures, hospital category and protocol in place

Table S6.1. Liberal (<2 hours), 2-4 hours and prolonged (>4 hours) rates for SIPS and NOT SIPS times across centres

Centre		SIPS <2h		NOT SIPS <2h		SIPS 2-4 h		NOT SIPS 2-4 h		SIPS >4h		NOT SIPS >4h	
		No	Yes	No	Yes	No	Yes	No	Yes	No	Yes	No	Yes
**1**	***n***	417	64	468	13	320	161	444	37	225	256	50	431
**(*n*=481)**	**%**	86.7%	13.3%	97.3%	2.7%	66.5%	33.5%	92.3%	7.7%	46.8%	53.2%	10.4%	89.6%
**2**	***n***	167	3	170	0	142	28	166	4	31	139	4	166
**(*n*=170)**	**%**	98.2%	1.8%	100.0%	0.0%	83.5%	16.5%	97.6%	2.4%	18.2%	81.8%	2.4%	97.6%
**3**	***n***	51	3	54	0	48	6	54	0	9	45	0	54
**(*n*=54)**	**%**	94.4%	5.6%	100.0%	0.0%	88.9%	11.1%	100.0%	0.0%	16.7%	83.3%	0.0%	100.0%
**4**	***n***	50	0	50	0	41	9	48	2	9	41	2	48
**(*n*=50)**	**%**	100.0%	0.0%	100.0%	0.0%	82.0%	18.0%	96.0%	4.0%	18.0%	82.0%	4.0%	96.0%
**5**	***n***	153	9	161	1	119	43	155	7	52	110	8	154
**(*n*=162)**	**%**	94.4%	5.6%	99.4%	0.6%	73.5%	26.5%	95.7%	4.3%	32.1%	67.9%	4.9%	95.1%
**6**	***n***	48	3	51	0	43	8	51	0	11	40	0	51
**(*n*=51)**	**%**	94.1%	5.9%	100.0%	0.0%	84.3%	15.7%	100.0%	0.0%	21.6%	78.4%	0.0%	100.0%
**7**	***n***	86	4	90	0	83	7	90	0	11	79	0	90
**(*n*=90)**	**%**	95.6%	4.4%	100.0%	0.0%	92.2%	7.8%	100.0%	0.0%	12.2%	87.8%	0.0%	100.0%
**8**	***n***	155	4	157	2	110	49	141	18	53	106	20	139
**(*n*=159)**	**%**	97.5%	2.5%	98.7%	1.3%	69.2%	30.8%	88.7%	11.3%	33.3%	66.7%	12.6%	87.4%
**9**	***n***	84	9	93	0	75	18	91	2	27	66	2	91
**(*n*=93)**	**%**	90.3%	9.7%	100.0%	0.0%	80.6%	19.4%	97.8%	2.2%	29.0%	71.0%	2.2%	97.8%
**10**	***n***	95	9	103	1	77	27	100	4	36	68	5	99
**(*n*=104)**	**%**	91.3%	8.7%	99.0%	1.0%	74.0%	26.0%	96.2%	3.8%	34.6%	65.4%	4.8%	95.2%
**11**	***n***	56	3	59	0	40	19	58	1	22	37	1	58
**(*n*=59)**	**%**	94.9%	5.1%	100.0%	0.0%	67.8%	32.2%	98.3%	1.7%	37.3%	62.7%	1.7%	98.3%
**12**	***n***	120	3	122	1	105	18	122	1	21	102	2	121
**(*n*=123)**	**%**	97.6%	2.4%	99.2%	0.8%	85.4%	14.6%	99.2%	0.8%	17.1%	82.9%	1.6%	98.4%
**13**	***n***	67	5	72	0	57	15	72	0	20	52	0	72
**(*n*=72)**	**%**	93.1%	6.9%	100.0%	0.0%	79.2%	20.8%	100.0%	0.0%	27.8%	72.2%	0.0%	100.0%
**14**	***n***	72	6	78	0	44	34	73	5	40	38	5	73

**(*n*=78)**	**%**	92.3%	7.7%	100.0%	0.0%	56.4%	43.6%	93.6%	6.4%	51.3%	48.7%	6.4%	93.6%
**15 (*n*=160)**	***n***	142	18	160	0	114	46	159	1	64	96	1	159
	**%**	88.8%	11.3%	100.0%	0.0%	71.3%	28.7%	99.4%	0.6%	40.0%	60.0%	0.6%	99.4%
**16 (*n*=64)**	***n***	63	1	64	0	55	9	63	1	10	54	1	63
	**%**	98.4%	1.6%	100.0%	0.0%	85.9%	14.1%	98.4%	1.6%	15.6%	84.4%	1.6%	98.4%
**17 (*n*=50)**	***n***	30	20	50	0	30	20	49	1	40	10	1	49
	**%**	60.0%	40.0%	100.0%	0.0%	60.0%	40.0%	98.0%	2.0%	80.0%	20.0%	2.0%	98.0%
**18 (*n*=50)**	***n***	50	0	50	0	45	5	45	5	5	45	5	45
	**%**	100.0%	0.0%	100.0%	0.0%	90.0%	10.0%	90.0%	10.0%	10.0%	90.0%	10.0%	90.0%
**19 (*n*=31)**	***n***	31	0	31	0	16	15	30	1	15	16	1	30
	**%**	100.0%	0.0%	100.0%	0.0%	51.6%	48.4%	96.8%	3.2%	48.4%	51.6%	3.2%	96.8%
**20 (*n*=34)**	***n***	34	0	34	0	25	9	34	0	9	25	0	34
	**%**	100.0%	0.0%	100.0%	0.0%	73.5%	26.5%	100.0%	0.0%	26.5%	73.5%	0.0%	100.0%
**21 (*n*=50)**	***n***	50	0	50	0	46	4	49	1	4	46	1	49
	**%**	100.0%	0.0%	100.0%	0.0%	92.0%	8.0%	98.0%	2.0%	8.0%	92.0%	2.0%	98.0%
**Total (*n*=2185)**	***n***	2021	164	2167	18	1635	550	2094	91	714	1471	109	2076
	**%**	92.5%	7.5%	99.2%	0.8%	74.8%	25.2%	95.8%	4.2%	32.7%	67.3%	5.0%	95.0%
Chi-square (χ^2), *p* value	**X^2** / ***p***	X^2 = 145,564, $p < 0.001$		X^2 = 30,86, $p = 0.057$		X^2 = 110,433, $p < 0.001$		X^2 = 67,826, $p < 0.001$		X^2 = 211,65, $p < 0.001$		X^2 = 86,343, $p < 0.001$	

Table S6.2. Liberal (<2 hours), 2-4 hours and prolonged (>4 hours) rates for SIPS and NOT SIPS times across specialties

Centre		SIPS <2h		NOT SIPS <2h		SIPS 2-4 h		NOT SIPS 2-4 h		SIPS >4h		NOT SIPS >4h	
		No	Yes	No	Yes	No	Yes	No	Yes	No	Yes	No	Yes
**Cardiothoracic**	***n***	34	0	34	0	24	10	32	2	10	24	2	32
**(*n*=34)**	**%**	100.0%	0.0%	100.0%	0.0%	70.6%	29.4%	94.1%	5.9%	29.4%	70.6%	5.9%	94.1%
**Endoscopy**	***n***	421	6	426	1	313	114	404	23	120	307	24	403
**(*n*=427)**	**%**	98.6%	1.4%	99.8%	0.2%	73.3%	26.7%	94.6%	5.4%	28.1%	71.9%	5.6%	94.4%
**ENT**	***n***	73	5	77	1	61	17	74	4	22	56	5	73
**(*n*=78)**	**%**	93.6%	6.4%	98.7%	1.3%	78.2%	21.8%	94.9%	5.1%	28.2%	71.8%	6.4%	93.6%
**General**	***n***	588	36	621	3	496	128	614	10	164	460	13	611
**(*n*=624)**	**%**	94.2%	5.8%	99.5%	0.5%	79.5%	20.5%	98.4%	1.6%	26.3%	73.7%	2.1%	97.9%
**Neurosurgery**	***n***	70	2	72	0	60	12	72	0	14	58	0	72
**(*n*=72)**	**%**	97.2%	2.8%	100.0%	0.0%	83.3%	16.7%	100.0%	0.0%	19.4%	80.6%	0.0%	100.0%
**NORA**	***n***	43	0	43	0	33	10	40	3	10	33	3	40
**(*n*=43)**	**%**	100.0%	0.0%	100.0%	0.0%	76.7%	23.3%	93.0%	7.0%	23.3%	76.7%	7.0%	93.0%
**Obstetrics-Gynecology**	***n***	206	19	224	1	165	60	220	5	79	146	6	219
**(*n*=225)**	**%**	91.6%	8.4%	99.6%	0.4%	73.3%	26.7%	97.8%	2.2%	35.1%	64.9%	2.7%	97.3%
**Ophthalmology**	***n***	88	51	128	11	82	57	113	26	108	31	37	102
**(*n*=139)**	**%**	63.3%	36.7%	92.1%	7.9%	59.0%	41.0%	81.3%	18.7%	77.7%	22.3%	26.6%	73.4%
**Orthopaedic surgery**	***n***	227	23	250	0	189	61	242	8	84	166	8	242
**(*n*=250)**	**%**	90.8%	9.2%	100.0%	0.0%	75.6%	24.4%	96.8%	3.2%	33.6%	66.4%	3.2%	96.8%
**Other**	***n***	32	4	36	0	30	6	31	5	10	26	5	31
**(*n*=36)**	**%**	88.9%	11.1%	100.0%	0.0%	83.3%	16.7%	86.1%	13.9%	27.8%	72.2%	13.9%	86.1%
**Plastic**	***n***	41	5	45	1	27	19	45	1	24	22	2	44
**(*n*=46)**	**%**	89.1%	10.9%	97.8%	2.2%	58.7%	41.3%	97.8%	2.2%	52.2%	47.8%	4.3%	95.7%
**Urology**	***n***	157	11	168	0	123	45	165	3	56	112	3	165
**(*n*=168)**	**%**	93.5%	6.5%	100.0%	0.0%	73.2%	26.8%	98.2%	1.8%	33.3%	66.7%	1.8%	98.2%
**Vscular**	***n***	41	2	43	0	32	11	42	1	13	30	1	42
**(*n*=43)**	**%**	95.3%	4.7%	100.0%	0.0%	74.4%	25.6%	97.7%	2.3%	30.2%	69.8%	2.3%	97.7%
**Total**	***n***	2021	164	2167	18	1635	550	2094	91	714	1471	109	2076
**(*n*=2185)**	**%**	92.5%	7.5%	99.2%	0.8%	74.8%	25.2%	95.8%	4.2%	32.7%	67.3%	5.0%	95.0%
**Chi-square (χ^2), *p* value**	**X^2** / ***p***	X^2 = 208,312, $p < 0.001$		X^2 = 95,224, $p < 0.001$		X^2 = 38,191, $p < 0.001$		X^2 = 104,344, $p < 0.001$		X^2 = 161,26, $p < 0.001$		X^2 = 167,792, $p < 0.001$	

Table S6.3. Liberal (<2 hours), 2-4 hours and prolonged (>4 hours) rates for SIPS and NOT SIPS times across hospital categories

Hospital category		SIPS <2h		NOT SIPS <2h		SIPS 2-4 h		NOT SIPS 2-4 h		SIPS >4h		NOT SIPS >4h	
		No	Yes	No	Yes	No	Yes	No	Yes	No	Yes	No	Yes
**Ia (*n*=367)**	***n***	351	16	364	3	276	91	343	24	107	260	27	340
	**%**	95.6%	4.4%	99.2%	0.8%	75.2%	24.8%	93.5%	6.5%	29.2%	70.8%	7.4%	92.6%
**Ib (*n*=1096)**	***n***	1011	85	1081	15	815	281	1045	51	366	730	66	1030
	**%**	92.2%	7.8%	98.6%	1.4%	74.4%	25.6%	95.3%	4.7%	33.4%	66.6%	6.0%	94.0%
**II (*n*=499)**	***n***	463	36	499	0	399	100	491	8	136	363	8	491
	**%**	92.8%	7.8%	100.0%	0.0%	80.0%	20.0%	98.4%	1.6%	27.3%	72.7%	1.6%	98.4%
**III (*n*=223)**	***n***	196	27	223	0	145	78	215	8	105	118	8	215
	**%**	87.9%	12.1%	100%	0.0%	65.0%	35.0%	96.4%	3.6%	47.1%	52.9%	3.6%	96.4%
**Chi-square (χ^2), *p* value**	**X^2** / ***p***	X^2 = 12,194, p = 0.007		X^2 = 9,979, p = 0.019		X^2 = 18,515, p < 0.001		X^2 = 14,230, p = 0.003		X^2 = 30,038, p < 0.001		X^2 = 19,802, p < 0.001	

Table S6.4. Liberal (<2 hours), 2-4 hours and prolonged (>4 hours) rates for SIPS and NOT SIPS times across protocol in place

Protocol in place		SIPS <2h		NOT SIPS <2h		SIPS 2-4 h		NOT SIPS 2-4 h		SIPS >4h		NOT SIPS >4h	
		No	Yes	No	Yes	No	Yes	No	Yes	No	Yes	No	Yes
**NPO after midnight (*n*=420)**	***n***	401	19	420	0	325	95	408	12	114	306	12	408
	**%**	95.5%	4.5%	100%	0.0%	77.4%	22.6%	97.1%	2.9%	27.1%	72.9%	2.9%	97.1%
**Fluids allowed until morning (*n*=690)**	***n***	650	40	686	4	514	176	665	25	216	474	29	661
	**%**	94.2%	5.8%	99.4%	0.6%	74.5%	25.5%	96.4%	3.6%	31.3%	68.7%	4.2%	95.8%
**Guidelines-based (*n*=1075)**	***n***	979	105	1061	14	796	279	1021	54	384	691	68	1007
	**%**	90.2%	9.8%	98.7%	1.3%	74.0%	26.0%	95.0%	5.0%	35.7%	64.3%	6.3%	93.5%
**Chi-square (χ^2), *p* value**	**X^2** / ***p***	X^2 = 16,202, p < 0.001		X^2 = 7,005, p = 0.03		X^2 = 1,843, p = 0.398		X^2 = 4,291, p = 0.117		X^2 = 10,966, p = 0.004		X^2 = 8,978, p = 0.011	
